# Unique growth pattern of human mammary epithelial cells induced by polymeric nanoparticles

**DOI:** 10.1002/phy2.27

**Published:** 2013-09-10

**Authors:** Rajaa Hussien, Bertrand H Rihn, Housam Eidi, Carole Ronzani, Olivier Joubert, Luc Ferrari, Oscar Vazquez, Daniela Kaufer, George A Brooks

**Affiliations:** 1 Department of Integrative Biology, University of CaliforniaBerkeley, California, 94720-3140; 2 Faculté de Pharmacie, EA 3452 CITHEFOR, Nancy-Université54001 Nancy Cedex, France; 3 Helen Wills Neuroscience Institute, University of CaliforniaBerkeley, California, 94720-3140

**Keywords:** Cell growth, cell proliferation, epithelial cells, Eudragit® RS nanoparticles, fetal bovine serum, nanoparticles

## Abstract

Due to their unique properties, engineered nanoparticles (NPs) have found broad use in industry, technology, and medicine, including as a vehicle for drug delivery. However, the understanding of NPs’ interaction with different types of mammalian cells lags significantly behind their increasing adoption in drug delivery. In this study, we show unique responses of human epithelial breast cells when exposed to polymeric Eudragit® RS NPs (ENPs) for 1–3 days. Cells displayed dose-dependent increases in metabolic activity and growth, but lower proliferation rates, than control cells, as evidenced in tetrazolium salt (WST-1) and 5-bromo-2′-deoxyuridine (BrdU) assays, respectively. Those effects did not affect cell death or mitochondrial fragmentation. We attribute the increase in metabolic activity and growth of cells culture with ENPs to three factors: (1) high affinity of proteins present in the serum for ENPs, (2) adhesion of ENPs to cells, and (3) activation of proliferation and growth pathways. The proteins and genes responsible for stimulating cell adhesion and growth were identified by mass spectrometry and Microarray analyses. We demonstrate a novel property of ENPs, which act to increase cell metabolic activity and growth and organize epithelial cells in the epithelium as determined by Microarray analysis.

## Introduction

The size, penetrative, carrier, and other properties of nanoparticles (NPs) allow them to enter the human body through inhalation, digestion, injection, and skin contact (Oberdorster et al. 2005; Rivera-Gil et al. 2012). Within the body, NPs can be transported to organs, tissues, and cells far from the site of exposure, where NPs cross anatomical barriers and penetrate live cells and their organelles (Rivera-Gil et al. 2012). The nanoparticulated forms of drugs and growth factors have potential to increase bioavailability, stability, and action of those agents while at the same time decrease toxicity and delivery variability associated with dosimetry (Santamaria 2012). Accordingly, among the diverse potential uses of NPs are their potential for targeted effects, including chemotherapy (Waite and Roth 2012). However, because the interactions between unloaded NPs and different types of mammalian cells are poorly understood we undertook this investigation.

Eudragit® RS NPs (ENPs) are a nonbiodegradable, positively charged copolymer (Eidi et al. 2010), and have been used for per os administration of drugs such as ibuprofen, cyclosporine, and indomethacin (Pignatello et al. 2002; Bhardwaj et al. 2010; Eidi et al. 2012). Recent work by Eidi et al. (2012) on the effect of unloaded ENPs on rat macrophages caused concern among researchers when results showed cytotoxic effects that included apoptosis, autophagy, and possible mitochondrial fragmentation. Concerns over toxicity of NP treatment emerged when studies demonstrated that some engineered NPs, including carbon nanotubes, titanium dioxide, and aluminum oxide, caused inflammatory responses in rat lungs. Similarly, carbon fullerenes have been shown to cause lipid peroxidation in the brain and gills of largemouth bass (Oberdorster 2001, 2004; Warheit et al. 2004; Santamaria 2012), and polyacrylate ester, one component of ENPs, was shown to cause pleural effusion, pulmonary fibrosis, and granuloma in humans (Song et al. 2009). Hence, in retrospect the effect of ENPs on macrophages in Eidi et al. may not be surprising because macrophages are designed to induce phagocytosis of living as well as inert materials, such as ENPs. Despite the expected behavior of macrophages, the study of Eidi et al. showed the need for further research to clarify the effect of unloaded ENPs on the growth of different types of cells before attempting drug delivery to cells, tissues, and organisms.

The few studies that have been performed with Eudragit or its constitutive polymers have produced contradictory results on its effect on cell growth. Eudragit RL stimulated the growth of fibroblasts (Boag and Sefton 1987), while Eudragit RS 100 slowed the growth of hepatocytes while activating their differentiation (Hamamoto et al. 1998). The constitutive polymers of Eudragit, poly(methylmethacrylate)-poly(methacrylic acid) copolymer, displayed no effect on the viability of neural cells (Dekeyser et al. 2008). However, none of the above studies used ENPs, nor did they report the effect of ENPs on cell metabolic activity or the growth of epithelial cells.

Given uncertainties about the effect of NPs, in general, and ENPs in particular, the goal of our study was to evaluate the effect of unloaded ENPs on the metabolic activity, growth, and proliferation of epithelial cells in culture. A range of 3–200 μg/mL of ENPs for 2000 cells per well of a 96-well plate was selected to compare our data to the data of Eidi et al., who used a similar range of ENP concentration. The use of different amounts of ENPs is important in our study, because it reflects the possibility of various actual direct and indirect exposure levels.

## Materials and Methods

### Preparation of ENPs

The Eudragit® RS 100 copolymer used in this study was purchased from Evonik Industries (Essen, Germany), and is a copolymer of ethylacrylate and methylmethacrylate, with low methacrylic acid ester content with quaternary ammonium groups. ENPs were prepared as described previously (Eidi et al. 2010) by dissolving a copolymer of ethylacrylate and methylmethacrylate in acetone (20 mg/mL). The organic solution was poured in a syringe, flowed under stirring in 40 mL of a Pluronic® F68 (Sigma, ST. Louis, MO) (0.5%, w/v) aqueous phase. The solvent was removed by rotary evaporation under vacuum at 40°C to a final polymer concentration of 7.5 mg/mL. The obtained NPs displayed a monodispersed distribution at 65.0 ± 26.3 nm and a Z-average of +51.04 mV. Their refractive index was 1.59, the viscosity was 0.8872 cP, and the relative density ranged from 0.816 to 0.836 (data not shown). The concentration of Pluronic F68 was 0.0008% for 200 μg/mL concentration of polymer. The potential effect of this concentration of Pluronic F68 on cells was evaluated, and none was observed (data not shown).

### Cell proliferation assay WST-1

Human breast cancer cell lines (MCF-7, MDA-MB-231) and the primary human breast cell line (HMEC 184) were grown in their respective media as described previously (Hussien and Brooks 2011). Cells were seeded at 2000 per well and grown at 37°C in an air/CO_2_ atmosphere (95/5 v/v) for 24, 48, and 72 h in the presence of 0, 3.1, 6.2, 12.5, 25, 50, 100, or 200 μg/mL ENPs in 200 μL of their respective media. At each time point, the medium was discarded, cells were washed with phosphate-buffered saline (PBS) 1×, and Dulbecco's Modified Eagle Medium without phenol red (supplemented with 10% FBS [fetal bovine serum], 1% l-glutamine, and 0.25% penicillin–streptomycin) but containing WST-1 (Cell Proliferation Reagent, Roche, Germany) was added and cells were incubated for 1–5 h. The 96-well plates were then read by spectrophotometry at 450 nm.

### BrdU and EdU cell proliferation assays

Cells were seeded in 96-well plates at 2000 per well and grown in a tissue culture incubator at 37°C and 5% CO_2_ for 24 h in the presence of ENPs in 200 μL of their respective media. 5-bromo-2′-deoxyuridine (BrdU) was used to label proliferating cells with a BrdU cell proliferation assay (Calbiochem, Darmstadt, Germany). Cells were incubated with BrdU for 6 h, then fixed with fixing/denaturing solution. The BrdU-labeled DNA was detected with a BrdU Mouse mAb kit using the manufacturer's protocol. The absorbance in each plate was measured using a spectrophotometer at dual wavelengths of 450–540 nm. To quantify the percentage of proliferating cells and total cell count after 24 h, HMEC 184 cells were seeded (5000 per chamber) in an eight-chamber slide (Lab-tek, PA) and treated with 0, 6, 25, and 100 μg/mL ENPs (Carlsbad, CA) for 24 h. Cells were incubated with 5-ethynyl-2′-deoxyuridine (EdU) (30 μmol/L) from Invitrogen (Carlsbad, CA) for 6 h, and then washed twice with PBS and fixed with 4% paraformaldehyde. Cells were permeabilized with 0.1% Triton-X 100 in PBS for 5 min, then incubated with 5% normal donkey serum-blocking buffer for 2 h. Incorporated EdU was detected with a copper-catalyzed fluorescent azide reaction (Click-iT; Invitrogen), after which slides were washed with PBS and mounted on cover slips with mounting medium containing 4′,6-diamidino-2-phenylindole (DAPI) (Vector, Burlingame, CA). Nuclei and EdU-positive nuclei were counted using the (20×/0.8 NA) air objective of an Axio Observer.Z1 fluorescence microscope (Zeiss, Germany) with Metamorph software (Molecular Devices, CA).

### Confocal laser scanning microscopy

ENPs were conjugated with Nile red (nominal diameter = 73 nm, zeta potential = +47, and polydispersity index = 0.34) according to a method described previously (Yoo et al. 2011). Nuclei were stained with Hoechst 33342 (Sigma, ST. Louis, MO), mitochondria were stained with MitoTracker Deep Red 633 (Invitrogen/Molecular Probes, Eugene, OR), and the membranes were stained with Wheat Germ Agglutinin Alexa Fluor 488 conjugate, WGA (Invitrogen). Cells were observed with a (20×/1.0 NA) water-dipping objective in a Zeiss LSM 780 microscope. A three-dimensional (3D) movie of HMEC 184 cells incubated for 3 days with Nile red-ENPs was generated using Imaris software (Bitplane, Zurich, Switzerland). ImageJ software was used to find ENPs localized with mitochondria (Wayne Rasband, NIH, Bethesda, MD). The average mitochondrial volume of single HMEC 184 cells stained with MitoTracker was measured using Imaris software from 3D images.

### Total protein measurement and immunoblots

Cells were washed twice with PBS and then solubilized with 5% NP-40. Total protein concentration was determined using a BCA protein assay kit (Pierce Biotechnology, Radford, IL). Western blotting was performed as previously described (Hussien and Brooks 2011). Primary antibodies used were rabbit anti-cytochrome oxidase subunit IV, mouse anti-β-actin, and mouse anti-voltage-dependent anion channel (VDAC) (Abcam, Cambridge, MA). Band intensity was quantified as previously described (Hussien and Brooks 2011).

### Purification and identification of serum proteins coated on ENPs with proteomic mass spectrometry

A mixture of serum and ENPs was centrifuged at 2500*g* for 10 min and the obtained pellet was washed twice with PBS and exposed to organic extraction with dichloromethane. The organic phase was examined on thin layer chromatography (TLC), and the aqueous phase was examined with UV/VIS spectrometry. To identify the proteins attached to ENPs, FBS (5.9 mL, ∼21.44 mg of proteins) containing 780 μg/mL of ENPs was centrifuged at 10,000*g* for 3.5 min. The pellet was washed twice with an equal volume of PBS 1×, then resuspended in 1 mL of either glycine•HCl (100 mmol/L, pH = 3), Tris•HCl/NaCl (50 mmol/L/5 mol/L, pH = 8), or guanidine thiocyanate (6 mol/L). The three samples were run on SDS (sodium dodecyl sulfate) gel electrophoresis and stained with Coomassie Brilliant Blue. Seven bands from the SDS gel of guanidine thiocyanate were examined with matrix-assisted laser desorption/ionization-time of flight (MALDI-TOF) mass spectrometry (MS), which identified 290 nonredundant proteins belonging to *Bos taurus*, and nine human contaminant cytokeratins. A total of 178 proteins were identified and analyzed for name of product in *B*. *taurus* (as they appeared in Unigene (http://www.ncbi.nlm.nih.gov/UniGene/), name of gene in *Homo sapiens* counterpart, name of human counterpart protein (http://www.uniprot.org/uniprot/), and plasma levels in human and InterPro domains if applicable (http://www.genecards.org/). A total of 69 proteins were cited only by their *B. taurus* name and were not included in data analysis because either (i) their relative abundance (RA) was very low (1–10), (ii) their identified peptides span less than 3% of the protein sequence, or (iii) they were isoforms of, or closely related to, already analyzed proteins. Proteins were ranked according to their (i) abundance (A), namely the ratio of spectrum count/length, and (ii) sequence coverage (SC), namely the percentage of the entire sequence that was expressed in the peptides found in trypsin hydrolysate. The RA was calculated as the ratio of the most abundant protein to the least abundant protein. The InterPro domains (http://www.ebi.ac.uk/interpro/) of 178 proteins were retrieved and were submitted to the STRING database (http://string-db.org/).

### Total RNA extraction and microarray analysis

Total RNA was extracted from HMEC 184 cells (50% and 90% confluence) incubated with 25 μg/mL ENPs for 24 h, and without incubation (control). The quality of RNA extracted with RiboPure kit (Ambion, Austin, TX) was determined with spectrophotometry and capillary electrophoresis, using RNA 6000 Nano® (Agilent 2100 Bioanalyser™, Santa Clara, CA). cDNA synthesis, cRNA synthesis, Cy3-dye labeling, and microarray hybridization were carried out using 100 ng of total RNA according to manufacturer protocol (One-Color Microarray-Based Gene Expression Analysis, version 6.6). Microarray slides (SurePrint G3 Human GE v2 8x60K, Agilent technologies) were scanned with an Agilent DNA microarray scanner. The acquisition, quantification of array images, and primary data analysis were performed using Agilent Feature Extraction Software. Data were first normalized with quantile method and stringent filtering criteria were next used to identify genes whose expression level was significantly changed, with a modified Student's *t*-test (*P* ≤ 0.001) and FC (fold change) ≥2.0. FC of mean of three replicates (for each ENP exposure and cell condition) on control were calculated. The selected genes display acceptable false discovery rate (<15%) according to Benjamini et al. (2001). The Database for Annotation, Visualization, and Integrated Discovery (DAVID; http://david.abcc.ncifcrf.gov) was then used to analyze and extract (i) relevant GO terms (http://godatabase.org), (ii) functions and expression data on Genecard (http://www.genecards.org), and (iii) known and predicted protein–protein interactions (http://string-db.org) for selected genes (da Huang et al. 2009). The raw data of our microarrays are available on http://www.ncbi.nlm.nih.gov/geo/, using the GSE45598, GSE45868, and GSE45869 access numbers.

### Statistical analysis

Testing for significant differences between groups at *P* < 0.05 was done either by the Student's *t-*test, one-way analysis of variance (ANOVA) with Tukey's post test (comparing all groups), or Dunnett's post test (comparing all groups vs. control) using RLPlot or Prism software.

## Results

### Dose-dependent increases in metabolic activity of epithelial cells exposed to ENPs

**Figure 1:**
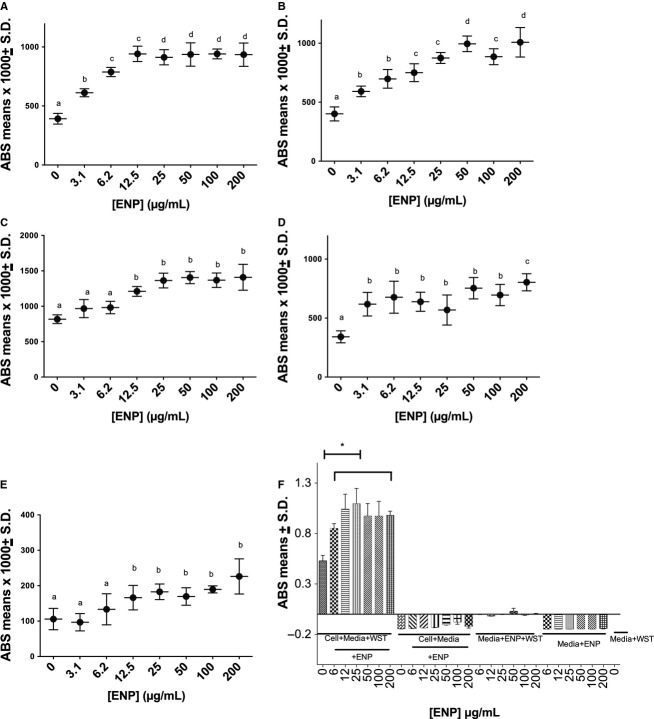
Metabolic activity of HMEC 184 cells (A, B, C), MDA-MB-231 (D), and MCF-7 (E) following 24 (A, D, E), 48 (B), and 72 h (C) exposure to various doses of ENPs (μg/mL). Different controls were tested in HMEC 184 cells to examine the effect of ENPs on the accuracy of the WST-1 assay (F). Data are means ± SD. Groups not sharing the same letter are different at the 95% level according to ANOVA analysis (*P* < 0.0001, Tukey's honest significant difference). *Significantly different (comparing all groups vs. control, Dunnett's post test).

**Figure A1:**
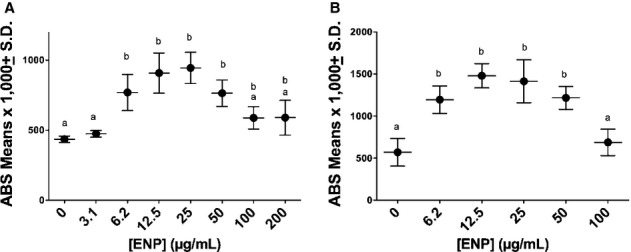
Metabolic activity of HMEC 184 cells following 48 h exposure to ENPs. ENPs (μg/mL) were preincubated for 24 h in plates with culture media at different concentrations before cells were seeded and incubated for 48 h (A). Cells were incubated for 48 h with ENPs (μg/mL) at different concentrations at the same time as cell seeding (B). Groups not sharing the same letter are different at the 95% level according to ANOVA analysis (*P* < 0.0001, Tukey's honest significant difference).

Human mammary epithelial cells (HMEC 184) (∼40% confluent) exposed to ENPs from 3.1 to 200 μg/mL for 24, 48, and 72 h showed a dose-dependent increase in metabolic activity, as measured by the WST-1 assay (Fig. 1A–C). The increase in WST-1 indicates an increase in the activity of mitochondrial succinate dehydrogenase (Mosmann 1983). Using HMEC 184 confluent cells (∼90% confluent), ENPs induced a dose-dependent increase in metabolic activity (data not shown). Two human epithelial breast cancer cell lines (MCF-7 and MDA-MB- 231) grown in different media were also incubated with varying doses of ENPs for 24 h; again, results showed a dose-dependent increase in metabolic activity (Fig. 1D and E). In a separate experiment, ENPs were mixed with culture media (3.1 to 200 μg/mL) and added to 96-well plates, either 24 h before seeding the HMEC 184 cells or at the same time as cell seeding, and a WST-1 assay was performed 2 days later. A similar trend of dose-dependent increased metabolic activity was seen in both cases (Fig. A1). Because a false-negative toxicity has been reported previously with 3-(4,5-dimethylthiazol-2yl)-2,5-diphenyltetrazolium bromide-formazan in interacting with NPs, but not with the WST-1 assay (Worle-Knirsch et al. 2006), additional control cells were subjected to WST-1 to rule out the possibility of reagent interaction with ENPs (Fig. 1F).

### Dose-dependent decrease in cell proliferation and cell count of epithelial cells exposed to ENPs

**Figure 2:**
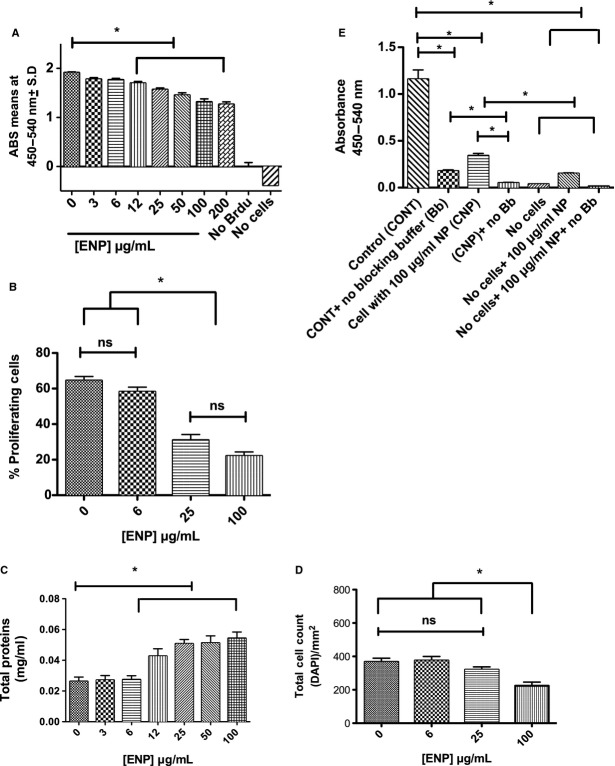
Cell proliferation (A, B), total proteins (C), and cell count (D) in HMEC 184 cells following 24 h exposure to ENPs (μg/mL). Cell proliferation (A) was measured with 5-bromo-2′-deoxyuridine (BrdU) incorporated into cellular DNA for 6 h, and a BrdU mouse mAb used to detect the BrdU-labeled DNA. A 5-ethynyl-2′-deoxyuridine (EdU) cell proliferation assay (B) demonstrated that ENPs decrease the percent of proliferation of HMEC 184 cells in culture in a dose-dependent manner. Different controls were tested in HMEC 184 cells to examine the effect of ENPs or blocking buffer on the accuracy of the BrdU assay (E). Data are means ± SD. *Significantly different at the 95% level according to ANOVA analysis (comparing all groups vs. control, Dunnett's post test).

**Figure A2:**
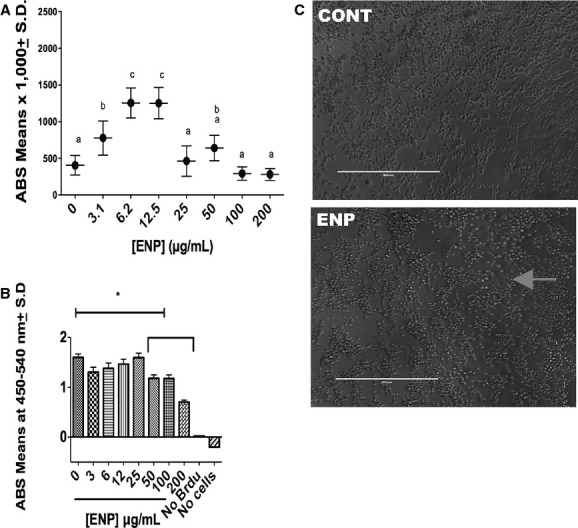
Metabolic activity (A) and cell proliferation (B) in neural progenitor cells (NPC) following 24 h exposure to ENPs (μg/mL). Metabolic activity and cell proliferation were measured with WST-1 and BrdU assays. A dose-dependent increase in metabolic activity and dose-dependent decrease in cell proliferation were seen in this cell line. ENPs formed a visible network with NPC media that adhered to cells in culture (C).

A dose-dependent decrease in cell proliferation, measured with a BrdU enzyme-linked immunosorbent assay (ELISA), was observed in the HMEC 184 cells after 24 h incubation with ENPs (Fig. 2A). The decrease in proliferation was further confirmed with a proliferation assay that quantified proliferating HMEC 184 cells labeled with EdU using a fluorescent azide reaction (Fig. 2B). An increase in total protein content in the HMEC 184 cells, measured with a BCA assay, was observed after 24 h of incubation with ENPs (Fig. 2C), and a decrease in total cell count was seen only in those cells incubated with a high dose of ENPs (100 μg/mL), as measured with DAPI stain (Fig. 2D). Additional control cells were subjected to BrdU assays to rule out the possibility of reagent interaction with ENPs (Fig. 2E). Neural progenitor cells (NPC) were treated with 0–200 μg/mL ENPs to test whether results were specific to epithelial cells. A similar dose-dependent increase in metabolic activity and dose-dependent decrease in cell proliferation were seen in those cells (Fig. A2).

### ENPs formed a visible network with serum proteins that adhered to cells in culture. ENPs entered the cells and caused an increase in total mitochondrial volume without an increase in mitochondrial biogenesis

**Figure 3:**
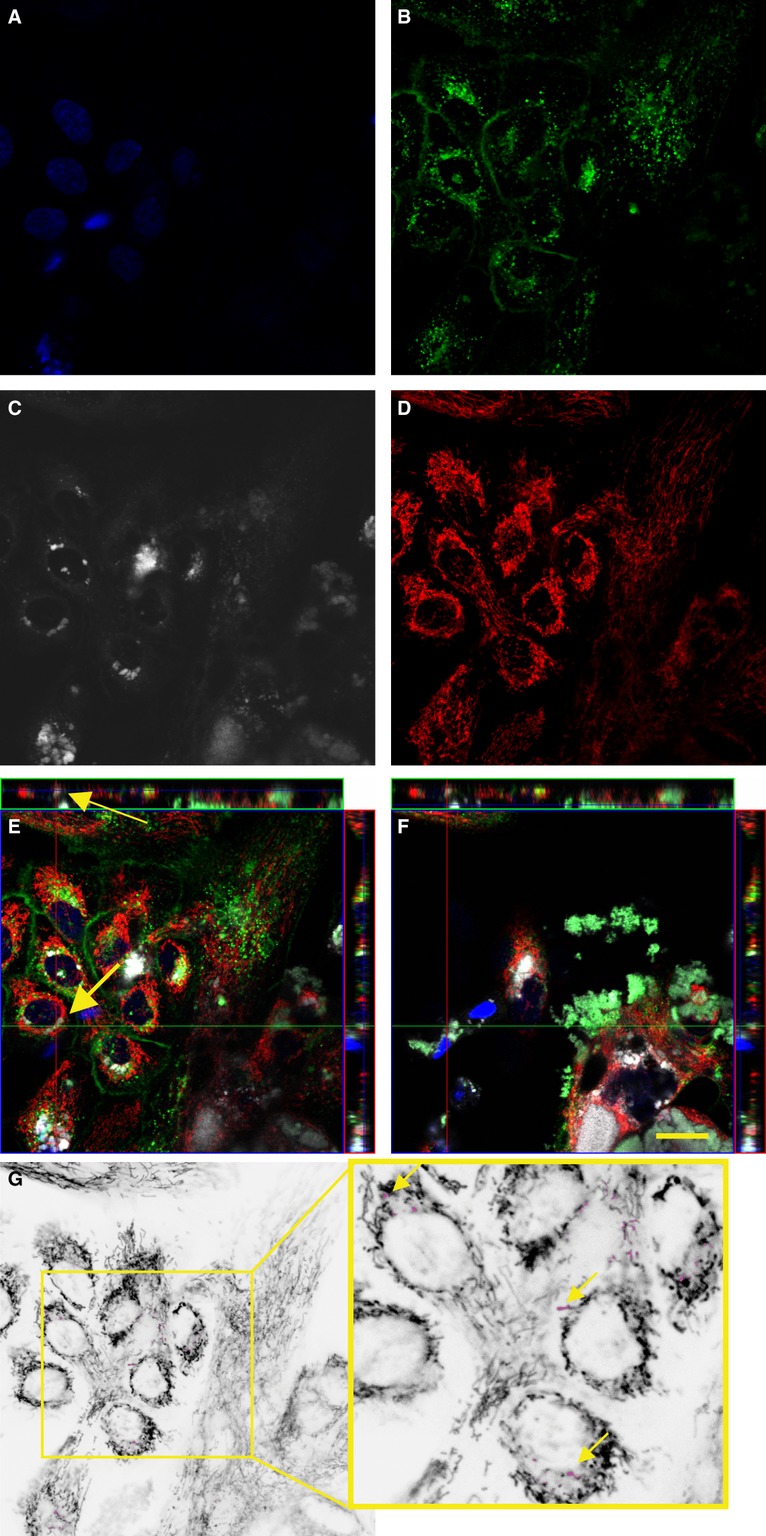
HMEC 184 cells after 3-day exposure to Nile red-ENP (25 μg/mL) as observed with a Zeiss LSM 780 confocal microscope. Nuclei were stained with 33342 Hoechst dye (A), membranes were stained with wheat germ agglutinin (B), ENPs were conjugated to Nile red (C), and mitochondria were stained with MitoTracker (D). Ortho-view of the z-stack shows that some ENPs are inside the cells (E) and some are aggregated on top of the cells (F). Colocalization analysis in ImageJ shows that some ENPs are colocalized with mitochondria (purple dot) (G). Whole images were contrast enhanced using ImageJ software. Scale bar = 20 μm.

**Figure 4:**
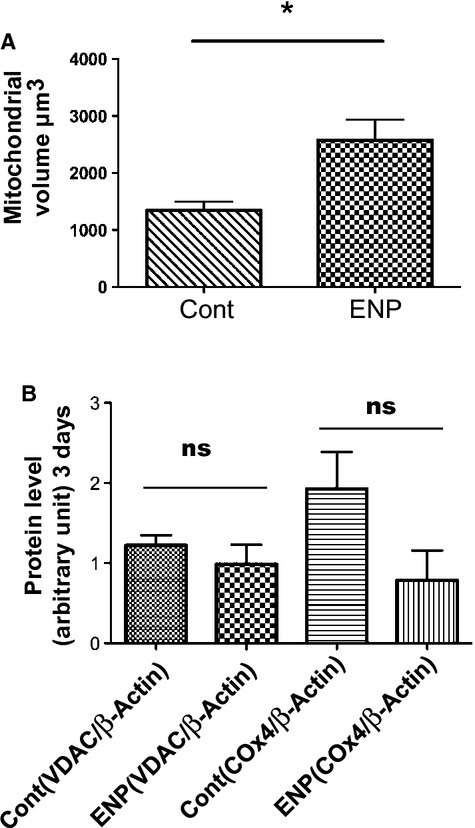
Mitochondrial volume (A) and protein content (B) in HMEC 184 cells after 3-day exposure to ENPs (25 μg/mL). Mitochondria were stained with MitoTracker, and mitochondrial volumes (μm^3^) were measured with Imaris software. There was an increase in mitochondrial volume in cells treated with ENPs as compared with control cells. A decrease in Cox4 and VDAC proteins was seen with Western blotting, but neither was a significant change. Data are means ± SD. *Significantly different at the 95% level according to ANOVA analysis (comparing all groups vs. control, Dunnett's post test).

An opalescent flocculate was visible when ENPs were added to HMEC 184, MDA-MB-231, and MCF-7 culture media. No flocculate was observed with the addition of ENPs to serum-free medium or PBS. Labeling of ENPs with Nile red fluorescent congregated dye and examination with confocal microscopy showed that ENPs entered the cells (Fig. 3 and Movie S1), but the majority of ENPs aggregated into clumps with proteins that formed a clearly visible network closely attached to cells. Colocalization analysis in ImageJ showed that some ENPs are localized with mitochondria (Fig. 3G). No fragmentation was seen in mitochondrial networks in HMEC 184, MDA-MB-231, and MCF-7 cells treated with ENPs at 25 μg/mL for 24 and 72 h (data not shown). There was an increase in the total mitochondrial volume in HMEC 184 cells treated with 25 μg/mL ENPs, when compared with untreated control cells as measured with Imaris software (Fig. 4A). However, a decrease in Cox4 and VDAC proteins was seen with Western blotting, but neither was a significant change (Fig. 4B). This implies an increase in cell size without a corresponding increase in mitochondrial biogenesis.

### Proteomic MS showed that the ENP-serum protein network contains proteins sharing common InterPro domains and exhibiting protease, antiprotease, epidermal growth factor, adhesion, and binding properties

To identify the nature of the visible flocculate, the pellet generated from centrifugation of a mixture of serum and ENPs was washed twice with PBS and exposed to organic extraction. No lipids were seen in the organic phase on TLC, but the aqueous phase showed a peak at 280 nm and an increased absorbance at 240 nm, with the UV/VIS spectrum suggesting the presence of proteins (data not shown). The pellet was examined with SDS-PAGE and MALDI-TOF MS, which identified 290 nonredundant proteins belonging to *B. taurus* and nine human cytokeratins. From MALDI-TOF MS, 178 proteins were identified and analyzed. Sequence coverage varied from 79.2% for albumin to 0.4% for titin. The RAs were calculated as the ratio of the most abundant to the least abundant protein, varied between 2581 and 1.

**Figure A3:**
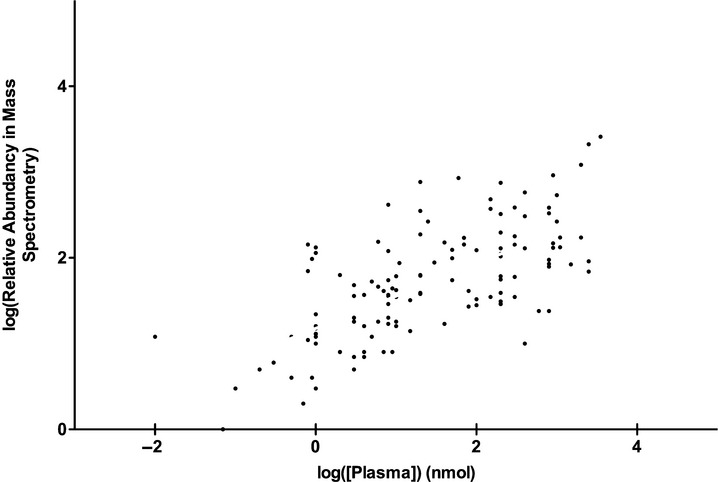
Linear regression analysis between the logarithm of plasma concentration (nmol) and the logarithm of relative abundance as obtained by mass spectrometry data. Regression analysis at a 95% confidence level showed a linear correlation between protein abundance in mass spectrometry and the concentration in plasma (*r* = 0.66, *P* < 0.0001).

**Figure A4:**
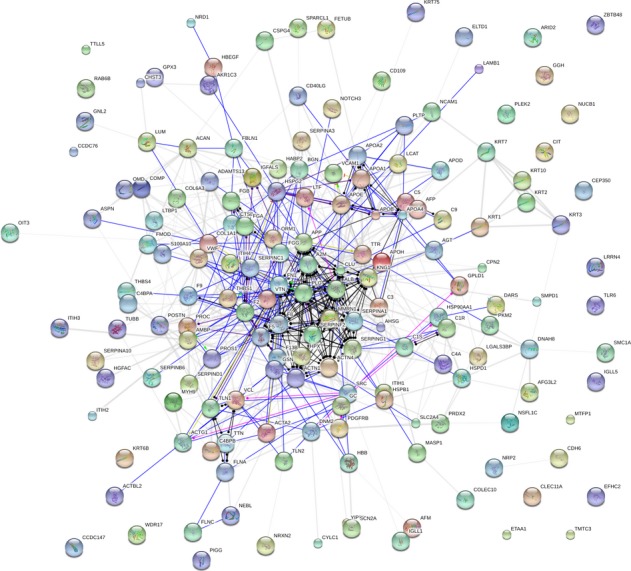
Interactions of purified gene products at the action level as retrieved from String. A total of 178 identified genes were submitted to the STRING database (http://string-db.org/). Of these, 169 genes were recognized by the STRING database and more than 80% showed interactions, either at the evidence, confidence, or action level.

Regression analysis at a 95% confidence level showed a linear correlation between protein abundance in MS and the protein concentration in plasma (Fig. A3). However, notable exceptions were seen later in the InterPro analysis. Because the coefficient of correlation (*r*) was only 0.66, and because some proteins were present at concentrations less than 1 nmol in plasma (e.g., kininogen-2 isoform II, actins, periostin, and serpinA 3-3), but were abundant in the MS chromatograms, we believe that proteins were not randomly fixed on ENPs according to their abundance in FBS. Therefore, we retrieved the InterPro domains of most of the 178 proteins we analyzed. The most frequently shared InterPro domain was “IPR006210:EGF-like,” shared by 18 different proteins (Table 1). Approximately 120 (70%) of 170 analyzed proteins shared at least two common domains. A high number of proteins belonging to InterPro domains are known to be involved in endopeptidase inhibitor activity, proteolytic activity or its regulation, protein binding, calcium binding, cell adhesion, and signaling, or have certain structural motifs like leucine-rich or immunoglobulin domains (Table 1). By calculating the mean of RA at the domain level, but not at the entire protein level, we found that proteins were coated not only due to their relative amounts in FBS but also due to their affinities for ENPs, reflecting their composition in domains rather than the structure of the entire protein (Table 1A). When submitting 178 genes to the STRING database, 169 protein encoding genes were recognized, and more than 80% of these showed interactions, either at evidence, confidence, or action levels. Seemingly, the purified proteins were also isolated by interactions between and among themselves (e.g., by protease inhibitor/protease interactions, or known binding properties of individual proteins). Serpins, proteases, and coagulation factors were found at the central core around which proteins involved in cell adhesion, growth, differentiation, and migration clustered (Fig. A4).

**Table 1:** Commonly shared domains of proteins purified by ENPs

InterPro Name	Number
IPR006210:EGF-like	18
IPR000215:Protease_inhib_14_serpin	13
IPR013783:Ig_like_fold	12
IPR016060:Complement_control_module	9
IPR001254:Peptidase_S1_S6	9
IPR001611:Leu-rich_rpt	9
IPR006209:EGF	9
IPR008985:ConA-like_lec_gl	8
IPR000436:Sushi_SCR_CCP	8
IPR009003:Pept_cys/ser_Trypsin-like	7
IPR018039:Intermediate_filament_CS	7
IPR011992:EF-hand-like_dom	6
PR002035:VWF_A	6
IPR001599:Macroglobln_a2	5
IPR000859:CUB	5
IPR008160:Collagen	5
IPR003961:Fibronectin_type3	5
IPR004001:Actin_CS	4
IPR000264:Serumumin	4
IPR000001:Kringle	4
IPR017857:Coagulation_fac_subgr_Gla_dom	4
IPR009053:Prefoldin	4
IPR018056:Kringle_CS	4
IPR000719:Prot_kinase_cat_dom	4
IPR001791:Laminin_G	4
IPR000010:Prot_inh_cystat	3
IPR000884:Thrombospondin_1_rpt	3
IPR012674:Calycin	3
IPR008979:Galactose-bd-like	3
IPR020837:Fibrinogen_CS	3
IPR001304:C-type_lectin	3
IPR002223:Prot_inh_Kunz-m	3
IPR017441:Protein_kinase_ATP_BS	3

The InterPro domains were retrieved from http://www.ebi.ac.uk/interpro/. About 120 of 170 analyzed proteins (70%) shared at least two domains.

### Microarray analysis for HMEC 184 cells treated with 25 μg/mL ENPs showed activation of proliferation and growth pathways

Microarray analysis was performed on HMEC 184 treated cells to identify the pathways induced by ENP treatment. HMEC 184 treated cells indicated 38 and 287 genes (50% and 90% confluence, respectively) whose expression was significantly altered when compared with control. The stringency of transcriptomic analysis was higher in the 90% confluence series (*P* < 0.001) than the 50% confluence series (*P* < 0.05). Few genes were downregulated in either cell culture condition: 3 and 4 genes for the 50% and 90% confluence series, respectively.

Several of the upregulated genes were genes involved in pathways of proliferation, growth, and transformation. These genes included *PIM-1*, which contributes to cell proliferation and survival; *VTCN1*, which promotes epithelial cell transformation; *ADRA1B* (upregulated in the 50% confluence dishes, data not shown), which activates mitogenic responses and has been found in normal and cancerous breast cell lines (Vazquez et al. 2006); *LCN2*, described as a gene involved in breast tumor progression (Yang et al. 2012); *ELF3*, an ETS domain transcription factor that is epithelial specific and is known to transactivate alone, or synergistically with other genes also upregulated in our experiment (such as *CLND7*, *FLG*, *KRT8*, *SPRR1A*, *MMP1*, *MM9*, and *TGM3*), epithelial cell differentiation; and *NDRG2*, which is involved in WNT signaling pathway (Shimomura et al. 2010; Lorentzen et al. 2011).

The functional annotation using DAVID revealed GO terms such as epithelial cell differentiation, epidermis development, response to wounding, and ectoderm development with a highly significant probability (*P* < 10E-12, FC > 10). These GO terms indicate that ENP action caused a spatial organization of breast epithelium (Table A2). The effect of ENP on epithelial cell organization was also seen in significant changes within genes involved in apicolateral plasma membrane organization, cell–cell junction, cell–cell adhesion, and apical junction complex organization. These genes included *CLDN4*, *CLDN3*, *CGN*, *CLDN7*, *DSG4*, and *CDSN*.

Differentially expressed genes at a level of *P* < 10E-03 were detected in breast tissues by Illumina Body Map (Cambridge, UK), indicating a strong relationship in expression pattern between HMEC 184 cell line and human breast tissue. Only nine of 84 genes (∼11%) displayed no expression in human breast tissue: *IL36RN*, *IL36G*, *CDSN*, *CWH43*, *ATP12A*, *NLRP10*, *IGFL2*, *KRT34*, and *MMP1*. Eighteen of the upregulated genes were described by Toulza et al. (2007) as upregulated genes in epidermal barrier function: *A2ML1*, *ADAM8*, *BNIPL*, *CDSN*, *CLDN3*, *CLDN4*, *CLDN7*, *DSG4*, *FLG*, *IGFL2*, *KLK6*, *KRT23*, *KRT24*, *KRT34*, *KRT80*, *LIPH*, *SERPINB2*, and *SPRR1A*.

Several genes upregulated in macrophages after exposure to NPs (Eidi et al. 2012) were also upregulated in HMEC 184 cells when exposed to ENPs. These genes were (i) *TLR1* and *2*, which recognize bacterial proteins or lipopeptides, and are activated by *IL8* (IL8 was also upregulated); (ii) *MARCO*, which binds to Gram (+) and (−) bacteria; and (iii) S100A12, which binds to ANXA1 (both were upregulated), and displays antibacterial activity against *Escherichia coli* and *Pseudomonas aeruginosa*. The upregulation of genes such as *RAB11FIP1*, *SPON2*, *MYO5B*, and *MAL2* indicates that the presence of ENPs with HMEC 184 cells allows those cells to acquire macrophage functions involved in endocytosis and potentializing MARCO.

Some of the induced genes code products that use proteins adsorbed on ENP as substrate, such as *MMP8*, *MMP9*, and *LCN2*, all of which activate procollagenase, and cleave collagene IV and VI, which bind to ENP. *MMP9* and *SERPINB2* degrade fibronectin and vitronectin, both adsorbed on ENP. *KK6* displayed hydrolytic activity against extracellular matrix proteins adsorbed on ENP, such as fibronectin, laminin, vitronectin, and collagen.

**Figure 5:**
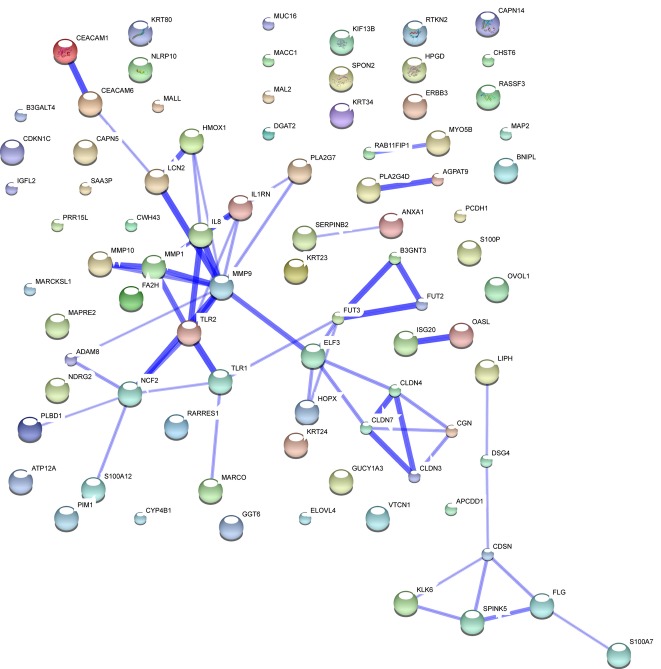
Protein–protein interactions of 84 overexpressed genes in HMEC 184 cells, as retrieved from String database. HMEC 184 cells (90% confluence) were incubated with and without 25 μg/mL ENPs for 24 h. Total RNA was extracted and analyzed with microarray. There were 287 genes whose expression was significantly altered when compared control. Forty-one of 84 genes recognized by String database were linked at the confidence, evidence, or action level.

Forty-one of the 84 genes in the 90% confluence series recognized by the STRING database (http://string-db.org) were linked at confidence, evidence, or action level (Fig. 5). Regarding the action level, some relevant catalysis involve: (i) initial activation of proMMP9 by MMP1, (ii) proMMP-9 activation by MMP-10, (iii) CEACAM1&6 heterodimer, (iv) MMP9 potentializing IL8, and (v) complex forming of serum amyloid A protein with upregulated TLR genes, SAA1/TLR2/TLR1.

## Discussion

We examined the effect of ENPs on metabolic activities and proliferation rates of epithelial in culture. We show here (1) an increase in metabolic activity and growth of epithelial cells after incubation with ENPs, (2) an activation of proliferation and growth genes in epithelial cells treated with ENPs, and (3) an effect of ENPs extracting from media and delivering to cell surfaces specific FBS plasma proteins involved in cell growth and metabolic activity of cells grown in culture. These results are discussed in more detail below.

### An increase in the metabolic activity and growth of epithelial cells after incubation with ENPs

Contrary to the results obtained by Eidi et al. (2012), who showed a cytotoxic effect of unloaded ENPs on macrophages, we show here no cytotoxicity effect of unloaded ENPs; in contrast, we show a dose-dependent increase in the metabolic activity of epithelial cells that was accompanied by a small, but significant dose-dependent decrease in cell proliferation. The overall effect was a small, but significant decrease in the total cell count when compared with ENP-untreated control cells. We attribute the increase in metabolic activity to an increase in cell size and total mitochondrial volume and activity. ENPs or similar products have been used by several groups on many type of cells for different reasons (Boag and Sefton 1987; Broughton and Sefton 1989; Hamamoto et al. 1998; Dekeyser et al. 2008; Eidi et al. 2012), but outcomes similar to the changes in metabolic activity and cell proliferation rate we found have not been reported. Differences may result from the use of different NPs or cell types by other groups. For example, ENPs used in this study were the same as those used by Eidi et al., so the differences in results are attributable to cell type. Macrophages are naturally designed to induce phagocytosis of inert or living particles, so it is not surprising that ENPs displayed cytotoxic effect on macrophages that were attributable to enhanced NP uptake and macrophage overload. In addition, the ENPs precipitated with FBS plasma proteins in the bottom of the culture dishes after ∼2 h, which may have reduced the amount of nutrients available for cells in suspension, such as macrophages, and may have increased the amount of nutrients available to adherent cells such as epithelial cells. Further research will be required to understand the contradictory results, but a similar trend was seen when neural stem cells were treated with ENPs, and so the newly described property of ENPs may affect multiple cell types.

### An activation of proliferation and growth genes in epithelial cells treated with ENPs

The observed effects of ENPs decreasing cell proliferation rates while increasing metabolic activity (measured with BrdU and WST-1, respectively) were unexpected. The microarray data showed an activation of proliferation and growth pathways in HMEC 184 cells treated with ENPs, but we only found an increase in growth and metabolic activity in those cells. Further research will be required to understand what prevented cell proliferation. It is possible that cells invested energy in metabolism and growth more than in proliferation. However, we saw no significant decrease in proliferation at lower levels of ENP treatment, which means that the lower ENP exposure has a positive effect on cell growth without an associated effect on proliferation. A similar result was obtained by Cui et al. (2012) who used gold NPs (Au NPs). Cui et al. showed that small Au NPs enter HeLa cells and cause a cytotoxic effect, but that large aggregated Au NPs adhere to the cell surface and cause an increase in cell growth.

Our data showed that ENPs caused an increase in cell metabolic activity in the three epithelial cells that were tested, the normal (HMEC 184) and the cancerous (MCF-7 and MDA-MB-231) cells. However, our results with the cancerous cells gives cause for concern regarding the use of ENPs as a cancer drug delivery system, because increasing the access of cancer cells to nutrients would be counterproductive even if ENPs targeted those cells and carried cytotoxic substances to them. On the other hand, stimulating growth in cells subjected to targeted chemotherapy could overstress normal cells leading to apoptosis. The effect of ENP treatment in increasing cancer cell metabolic activity has not been reported previously, and further research is needed to address the potentially counter-productive effect of ENP increasing cancer cell activity.

### ENP extracts and delivers to cell surfaces specific FBS plasma proteins that are crucial for cell growth and metabolic activity of cells grown in culture

At present it is premature to speculate about the mechanisms by which Eudragit RS served to increase metabolism in cells. However, through its dual function of “polyaffinity nanochromatography” (i.e., aggregating numerous factors that promote metabolism and growth) and adherence to cells, Eudragit RS in effect concentrates stimuli for cell growth on cell surfaces.

To date, other NPs, such as magnetic NPs coated with antibodies, ligands, or receptors, have been used to extract proteins from sera and other media (Safarik and Safarikova 2004; Gao et al. 2009). Eudragit RS 100 was reported by some teams for the purification of proteins from bacteria and yeast (such as protein A [Kamihira et al. 1992], β-glucosidase [Agarwal and Gupta 1996], and xylanase [Sharma and Gupta 2002]), the immobilization of enzymes (such as amylosucrase of *Nesseira* [Wang et al. 2011]), and in affinity chromatography for monoclonal antibody purification (Taipa et al. 2000). Eudragit RS 100 was also used by some groups to refold fibroblast growth factor (FGF) and lysozyme (Huang and Leong 2009). The same proposed mechanism was also reported with TGF-β and KGF-2 (Huang et al. 2009). Interestingly, the proteins extracted by the process described by those groups belong to an InterPro Domain, “TGF-β,” that is closely related to the protein TGF-β that refolds well in an Eudragit buffer.

Sharma and Gupta (2002) were the first to use the term “macroaffinity,” and to link it to use of the Eudragit RS 100 polymer. Following their lead we named our method “polyaffinity nanochromatography.” The methods we developed here allow the isolation of proteins that are closely related by structure, activity, and interactions, and that are not randomly distributed. In addition, the protocol we describe may allow the extraction and isolation of a select group of proteins for diagnostic purposes from animal and human fluids (serum, plasma, cerebrospinal fluid, urine, exudates, and transudates), as the proteins described here may also be present in those fluids in physiological or pathological conditions. Hence, the 45 proteins highlighted in were either never described in plasma or are present in plasma in pathological conditions. On the other hand, further research is required if ENPs are to be used increasingly in drug delivery, especially in light of their absorption of many important proteins and growth factors.

## Conclusion

Our data show that ENPs can be used to increase the metabolic activity and growth of epithelial cells in a dose-dependent manner. The mechanism for this behavior stems from the ability of ENPs to bind to certain proteins in culture media and to bring them closer to the surface of cells. Those proteins are involved in cell adhesion, growth, differentiation, and migration. The observed behaviors of ENPs suggest new uses of ENPs beyond drug coating.

## Appendix

**Table A1:** Relative abundance at the domain level

IP_Number	IP_Name	Protein function (gene ontology)	Number	Mean of domain RA
“IPR000215”	Protease_inhib_l4_serpin	Serine-type endopeptidase inhibitor activity (GO)	13	51
“IPR002035”	VWF_A	Protein binding (GO)	6	53
“IPR001599”	Macroglobln_a2	Endopeptidase inhibitor activity (GO)	5	59
“IPR018039”	lntermediate_filament_CS	Cytoskeleton structure	7	71
“IPR016060”	Complement_control_module	Protein binding	9	75
“IPR013806”	Kringle-like	Regulation of proteolytic activity	5	77
“IPR009003”	Pept_cys/ser_Trypsin-like	(Protease) catalytic activity (GO)	7	78
“IPR003591”	Leu-rich_rpt_typical-subtyp	LRR proteins	6	78
“IPR000372”	LRR-contain N	N-terminal LRR	7	82
“IPR001254”	Peptidase_S1_S6	Serine-type endopeptidase activity (GO)	9	84
“IPR000859”	CUB	MEROPS peptidase	5	91
“IPR001881”	EGF-like_Ca-bd	Calcium ion binding (GO)	14	92
“IPR001611”	Leu-rich_rpt	LRR proteins	9	95
“IPR011992”	EF-hand-like_dom	Calcium ion binding (GO)	6	97
“IPR013032”	EGF-like_reg_C	–	17	98
“IPR006210”	EGF-like	Protein binding (GO)	18	107
“IPR008985”	ConA-like_lec_gl	Adhesion	8	108
“IPR013320”	ConA-like_subgrp	Lectin	8	119
“IPR008160”	Collagen	adhesion	5	125
“IPR006209”	EGF	Protein binding (GO)	9	126
“IPR013783”	lg-like_fold	–	12	127
“IPR011993”	PH_type	Signalling	5	127
“IPR007110”	Ig-like	Protein binding (GO)	8	130
“IPR003961”	Fibronectin_type3	Protein binding(GO)	5	141
“IPR013098”	IgJ-set	Adhesion	5	155

A list of selected frequently appearing InterPro domains (IP number and IP name), their function as accepted and annotated by Gene Ontology, the number of different proteins sharing them, and their mean of relative abundance.

**Table A2:** Functional annotation analysis of microarray data sets using DAVID

Cluster	Category	Term	Count	*P*-value^1^	FE
Cluster 1	GOTERM_CC_FA T	Cornified envelope	9	1.78E-11	41.83
	SP_PIR_KEYWO RDS	Keratinization	7	6.27E-07	22.29
	GOTERM_BP_FA T	Keratinocyte differentiation	12	8.22E-12	21.02
	GOTERM_BP_FA T	Epidermal cell differentiation	13	8.83E-13	20.88
	GOTERM_BP_FA T	Keratinization	7	1.64E-06	18.82
	GOTERM_BP_FA T	Peptide cross-linking	4	1.38E-03	17.79
	GOTERM_BP_FA T	Epithelial cell differentiation	16	6.41E-13	13.50
	GOTERM_BP_FA T	Epidermis development	18	2.81E-13	11.31
	GOTERM_BP_FA T	Ectoderm development	18	1.02E-12	10.46
	GOTERM_BP_FA T	Epithelium development	16	9.11E-10	8.15
Cluster 2	SP_PIR_KEYWO RDS	Inflammatory response	5	3.09E-03	8.27
	GOTERM_BP_FA T	Inflammatory response	10	1.91E-03	3.56
	GOTERM_BP_FA T	Response to wounding	15	1.74E-04	3.27
	GOTERM_BP_FA T	Defense response	12	1.64E-02	2.26
Cluster 3	INTERPRO	Keratin_type I	3	2.80E-02	11.39
	INTERPRO	Filament	4	1.95E-02	6.96
	INTERPRO	Intermediate filament protein_conserved site	4	1.95E-02	6.96
	GOTERM_MF_FA T	Structural molecule activity	14	1.24E-03	2.81
Cluster 4	GOTERM_CC_FA T	Desmosome	3	1.58E-02	15.34
	SP_PIR_KEYWO RDS	Tight junction	3	8.21E-02	6.26
	GOTERM_CC_FA T	Apical junction complex	6	2.74E-03	6.20
	GOTERM_CC_FA T	Apicolateral plasma membrane	6	3.12E-03	6.02
	GOTERM_CC_FA T	Cell-cell junction	8	2.50E-03	4.31
Cluster 5	SP_PIR_KEYWO RDS	Inflammatory response	5	3.09E-03	8.27
Cluster 6	KEGG_PATHWA Y	Glycosphingolipid biosynthesis	3	1.12E-02	17.95
	SP_PIR_KEYWO RDS	Signal-anchor	10	5.85E-03	3.03
Cluster 7	GOTERM_BP_FA T	Glycerolipid biosynthetic process	4	3.13E-02	5.78
Cluster 8	GOTERM_BP_FA T	Wound healing	6	2.43E-02	3.63

GO terms were significantly enriched in genes at least twofold upregulated in HMEC 184 cells (90% confluence) in response to 24 h exposure to 25 µg/mL ENPs. Biological process (BP), cellular component (CC), and molecular function (MF) in Gene Ontology (GO), single protein of protein information ressource (SP_PIR), protein domains or sites (INTERPRO) and pathway extracted from Kyoto Encyclopedia of Genes and Genomes (KEGG).

1 EASE score, modified Fisher's exact test according to DAVID software cut-off.

## Supporting Information

**Movie S1.** A 3D movie of HMEC 184 cells after 3-day exposure to ENPs (25 μg/mL) as observed with a Zeiss LSM 780 confocal microscope. Nuclei were stained with Hoechst (blue), membranes were stained with wheat germ agglutinin (green), ENPs were conjugated to Nile red (white), and mitochondria were stained with MitoTracker Deep Red (red). ENPs were observed inside the cells and some were aggregated atop of the cells. https://docs.google.com/file/d/0B6TwZrW3wcudbkVaTklLd0U4eVE/edit?usp=sharing
