# Supplementary material for: Unique growth pattern of human mammary epithelial cells induced by polymeric nanoparticles
Source: Physiol Rep. 2013 Sep 10;1(4):e00027. doi: 10.1002/phy2.27 (PMC3831889; doi:10.1002/phy2.27)
Supplement: Supplementary file 1 [file phy20001-e00027-SD1.pdf]

Supplementary Figure 1. Metabolic activity of HMEC 184 cells following 48h exposure to ENPs. ENPs (µg/mL) were pre-incubated for 24h in plates with culture media at different concentrations before cells were seeded and incubated for 48h (a). Cells were incubated for 48h with ENPs (µg/mL) at different concentrations at the same time as cell seeding (b). Groups not sharing the same letter are different at the 95% level according to ANOVA analysis ($p < 0.0001$, Tukey's honest significant difference).

a

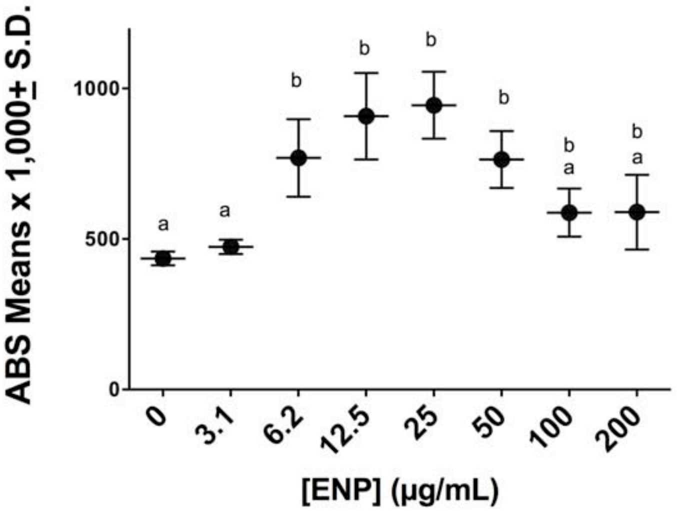

b

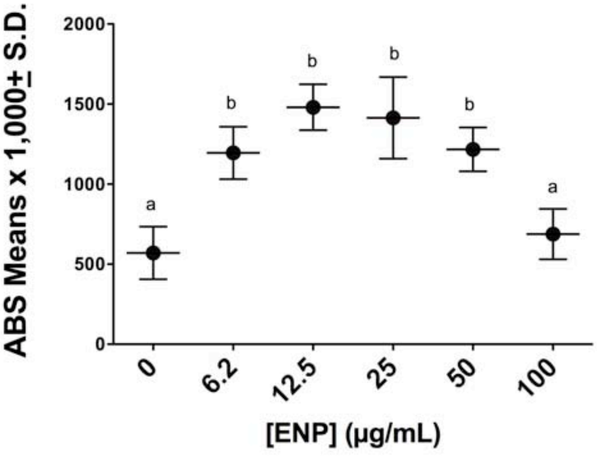

Supplementary Figure 2. Metabolic activity (a) and cell proliferation (b) in neural progenitor cells (NPC) following 24h exposure to ENPs (µg/mL). Metabolic activity and cell proliferation were measured with WST-1 and BrdU assays. A dose-dependent increase in metabolic activity and dose-dependent decrease in cell proliferation were seen in this cell line. ENPs formed a visible network with NPC media that adhered to cells in culture (c).

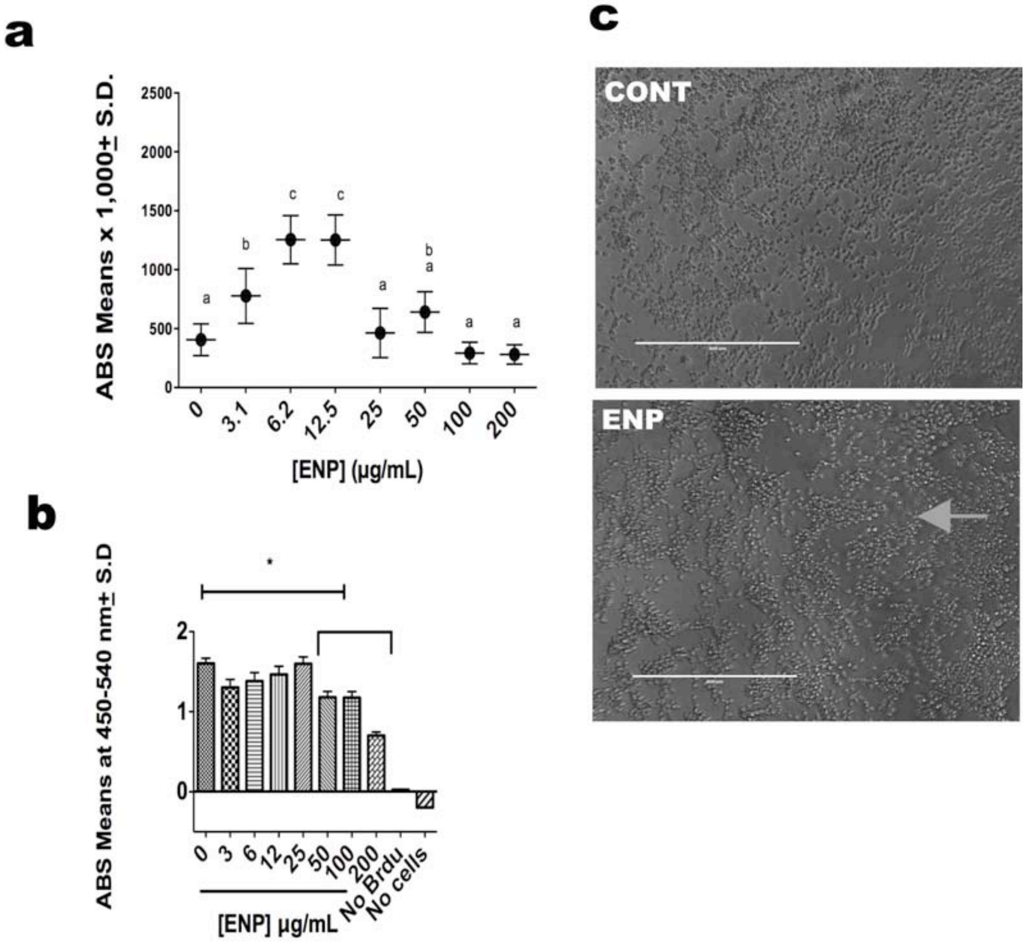

Supplementary Movie 1. A 3D movie of HMEC 184 cells after 3-day exposure to ENPs (25 µg/mL) as observed with a Zeiss LSM 780 confocal microscope. Nuclei were stained with Hoechst (blue), membranes were stained with wheat germ agglutinin (green), ENPs were conjugated to Nile red (white), and mitochondria were stained with MitoTracker Deep Red (red). ENPs were observed inside the cells and some were aggregated atop of the cells. https://docs.google.com/file/d/0B6TwZrW3wcudbkVaTklLd0U4eVE/edit?usp=sharing

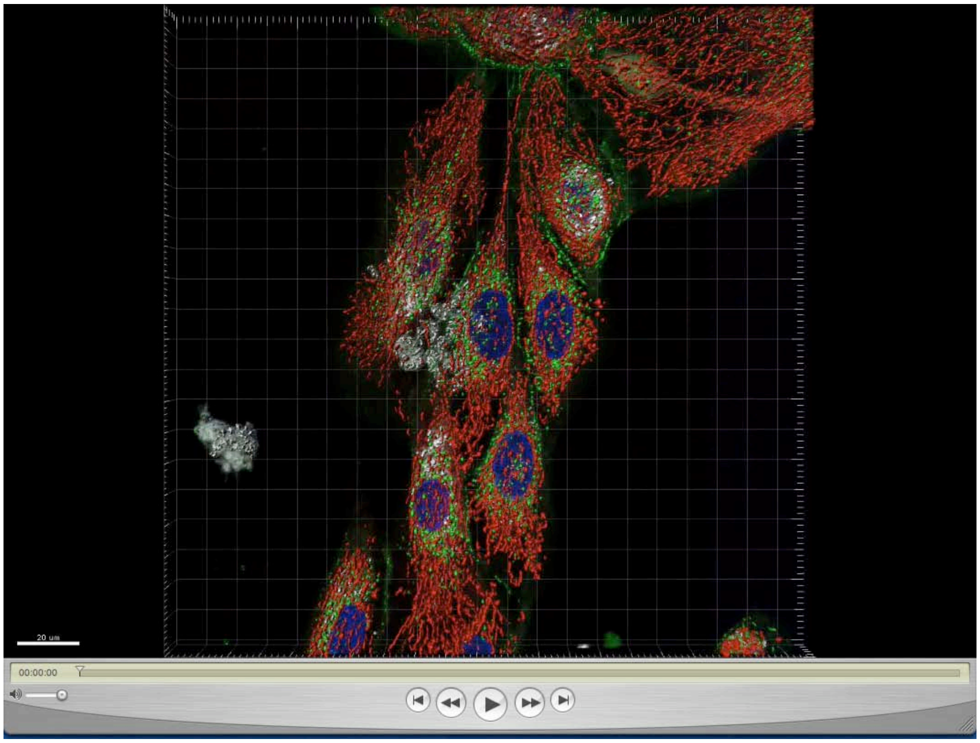

Supplementary Table 1[1]. A list of proteins from FBS purified by ENPs and identified with MALDI-TOF mass spectrometry. Mass spectrometry analysis identified ~299 proteins from FBS that were attached to ENPs. Of these, 178 proteins are listed here and were identified and analyzed for: name of product in *Bos taurus* (as appeared in Unigene), name of gene in *Homo sapiens* counterpart, name of human counterpart protein, and levels in human plasma. A notable fraction of those proteins (~45, highlighted in yellow) were either never described in plasma or are present in plasma in pathological conditions. Proteins are ranked according to their abundance (RA) and sequence coverage (SC). Sequence coverage varied from 79.2% for albumin to 0.4% for titin.

"GENE_ID "	"PROT_ID"	"Name"	"A"	"[Plasma] "	"RA "	"Rank RA"	"Rank SC"
ALB	P02768	serum albumin	0.9259	3500	2581.0	1	1
APOA1	P02647	apolipoprotein A-I	0.7585	2500	2114.4	2	4
SERPINA 1	P01009	alpha-1-antiproteinase	0.4351	2000	1212.9	3	16
SERPINC 1	P01008	antithrombin-III	0.3290	900	917.2	4	10
ITIH3	Q06033	inter-alpha-trypsin inhibitor heavy chain H3	0.3064	60	854.1	5	9
ACTA2	P62736	actin alpha cardiac muscle	0.2747	20	765.7	6	3
HBB	P68871	hemoglobin fetal subunit beta	0.2690	200	749.8	7	2
ITIH2	P19823	inter-alpha-trypsin inhibitor	0.2072	400	577.6	8	25

[1] "GENE_ID" : Gene ID of the human counterpart as found in Genecard,
"PROT_ID" : Protein ID of the human counterpart as found in the Uniprot database,
"Name" : Name of the protein in *Bos taurus*,
"A" : Abundancy in mass spectrometry = spectrum count/length,
"[Plasma]" : Plasma level (nmol),
"RA " : Relative Abundancy,
"Rank RA" : Rank by Relative Abundancy,
"Rank SC" : Rank by Sequence Coverage,
ND : Non Determined in Genecard database.
SC : The percentage of the entire sequence that was expressed in the peptides found in trypsin hydrolysate.

		heavy chain H2					
A2M	P01023	alpha-2-macroglobulin	0.1927	1000	537.2	9	5
LUM	P51884	lumican	0.1725	150	480.9	10	12
ACTBL2	Q562R1	actin beta-like 2	0.1489	8	415.2	11	50
C4BPA	P04003	C4b-binding protein alpha chain	0.1385	300	386.0	12	8
F2	P00734	prothrombin	0.1376	800	383.6	13	45
HBA	P69905	hemoglobin subunit alpha	0.1338	150	373.0	14	7
ACTG1	P63261	actin gamma-enteric smooth muscle	0.1277	0	355.9	15	19
LGALS3BP	Q08380	galectin-3-binding protein	0.1261	20	351.6	16	23
ACTA2L	nd	uncharacterized protein LOC782051	0.1228	0	342.3	17	54
KNG2	P01045	kininogen-2 isoform II	0.1228	0	342.3	18	17
APOA4	P06727	apolipoprotein A-IV	0.1184	800	330.1	19	6
AMBP	P02760	protein AMBP	0.1165	200	324.7	20	38
VTN	P04004	vitronectin	0.1092	400	304.5	21	57
GC	P02774	vitamin D-binding protein	0.0949	1000	264.7	22	18
FBLN1	P23142	fibulin-1	0.0949	25	264.6	23	34
SERPIND1	P05546	heparin cofactor 2	0.0706	200	196.7	24	37
SPARCL1	Q14515	SPARC-like protein 1	0.0673	20	187.5	25	11
CLU	P10909	clusterin	0.0638	300	177.8	26	21

		prepropr otein					
C3	P01024	complem ent C3 prepropr otein	0.0620	1100	172.9	27	13
SERPINA 3	P01011	plasma serine protease inhibitor	0.0619	2000	172.5	28	28
C4BPB	P20851	uncharac terized protein LOC5108 60	0.0612	70	170.7	29	31
IGLL1	P15814	immunog lobulin lambda-like polypepti de 1	0.0553	6	154.2	30	30
FETUB	Q9UGM5	fetuin-B	0.0543	40	151.3	31	35
AHSG	P02765	alpha-2-HS-glycoprot ein	0.0529	900	147.5	32	48
F13B	P05160	coagulati on factor XIII B polypepti de	0.0514	70	143.4	33	33
F8	P00451	coagulati on factor XIII B chain	0.0514	0.8	143.4	34	36
SERPINF 2	P08697	alpha-2-antiplasm in	0.0508	300	141.6	35	27
SERPINA 3_3	nd	serpin A3-3	0.0487	0	135.7	36	58
TTR	P02766	transthyr etin	0.0476	1100	132.7	37	14
POSTN	Q15063	periostin	0.0475	1	132.4	38	15
TRFE	P02787	serotrans ferrin	0.0469	900	130.7	39	20
ITIH1	P19827	inter-alpha-trypsin inhibitor heavy chain H1	0.0464	400	129.2	40	22
KRT1	P04264	keratin type II cytoskele	0.0462	200	128.8	41	94

		tal 1					
KRT2	P35908	keratin type II cytoskeletal 2	0.0446	50	124.2	42	102
KRT10	P13645	keratin type I cytoskeletal 10	0.0442	100	123.1	43	81
PLTP	P55058	phospholipid transfer protein	0.0431	8	120.3	44	46
SERPINA3_5	A2I7N1	serpin A3-5	0.0414	0	115.3	45	72
CD109	Q6YHK3	CD109 antigen	0.0408	1	113.8	46	39
AFM	P43652	afamin	0.0397	200	110.8	47	41
CD40LG	P29965	CD40 ligand	0.0383	0	106.8	48	124
APOB	P04114	apolipoprotein B-100	0.0373	200	104.1	49	32
GPX3	P22352	glutathione peroxidase	0.0354	50	98.7	50	44
FMOD	Q06828	fibromodulin	0.0347	0.9	96.6	51	53
FGA	P02671	fibrinogen alpha chain	0.0341	800	95.2	52	64
CLEC11A	Q9Y240	C-type lectin domain family 11 member A	0.0340	0	94.6	53	29
HPX	P02790	hemopexin	0.0327	2500	91.1	54	43
TLN1	Q9Y490	talin-1	0.0327	0	91.1	55	26
THBS1	P07996	thrombospondin-1	0.0316	30	88.2	56	40
PROC	P04070	vitamin K-dependent protein C	0.0313	11	87.1	57	52
ITIH4	Q14624	inter-alpha-trypsin inhibitor heavy chain H4	0.0306	800	85.2	58	55

APOA2	P02652	apolipoprotein A-II	0.0300	1500	83.6	59	42
FGB	P02675	fibrinogen beta chain	0.0283	800	78.8	60	86
HVM63	P84751	Ig heavy chain Mem5-like partial	0.0263	0	73.4	61	74
CHST3	Q7LGC8	carbohydrate sulfotransferase 3	0.0251	0.8	69.8	62	70
ORM1	P02763	alpha-1-acid glycoprotein	0.0248	2500	69.0	63	79
NRP2	O60462	neuropilin-2	0.0227	2	63.2	64	67
GPLD1	P80108	phosphatidylinositol-glycan-specific phospholipase D precurso	0.0226	20	63.1	65	47
SERPINA10	Q9UK55	protein Z-dependent protease inhibitor	0.0221	20	61.7	66	49
HSP90AA1	P07900	heat shock protein HSP 90-alpha	0.0218	10	60.8	67	68
FN1	P02751	fibronectin	0.0218	200	60.7	68	56
SERPINA3_8	A6QPQ2	SERPINA3-8	0.0215	0	60.0	69	164
IGLL5	B9A064	immunoglobulin lambda-like polypeptide 5-like	0.0214	300	59.6	70	78
S100A10	P60903	protein S100-A10	0.0206	0	57.5	71	99
C1S	P09871	complement C1s subcomponent	0.0201	200	56.2	72	93

TSP4	P35443	thrombospondin-4	0.0198	8	55.1	73	59
MTFP1	Q9UDX5	mitochondrial fission 1 protein	0.0197	0	55.0	74	51
HABP2	Q14520	hyaluronan-binding protein 2	0.0197	50	55.0	75	98
F12AI/ SERPING 1	P50448	factor XIIa inhibitor (~SERPING1)	0.0192	700	53.6	76	61
OMD	Q99983	osteomodulin	0.0190	5	52.8	77	113
KRT3	P12035	keratin type II cytoskeletal 3	0.0173	3	48.3	78	108
HSPB1	P04792	heat shock protein HSP 90-beta	0.0166	6	46.2	79	76
SMPD1	P17405	sphingomyelin phosphodiesterase	0.0160	0	44.6	80	89
VWF	P04275	von Willebrand factor	0.0157	9	43.7	81	97
CTSB	P07858	cathepsin B	0.0149	10	41.6	82	65
AFP	P02771	alpha-fetoprotein	0.0148	0	41.1	83	66
CPN2	P22792	carboxypeptidase N subunit 2	0.0146	80	40.7	84	95
COMP	P49747	cartilage oligomeric matrix protein	0.0146	7	40.6	85	71
PLEK2	Q9NYT0	pleckstrin	0.0143	0	39.8	86	73
AGT	P01019	angiotensinogen	0.0142	200	39.4	87	62
HGFAC	Q04756	hepatocyte growth factor activator	0.0138	20	38.5	88	84

		preproprotein					
MASP1	P48740	mannan-binding lectin serine protease 1	0.0137	20	38.3	89	60
LCAT	P04180	phosphatidylcholine-sterol acyltransferase	0.0136	20	38.0	90	88
TUBB	P07437	tubulin beta-1 chain	0.0133	4	37.2	91	83
SERPINB6	P35237	serpin B6	0.0132	8	36.9	92	106
LTBP1	Q14766	latent-transforming growth factor beta-binding protein 4-like	0.0129	8	36.0	93	87
KRT75	O95678	keratin type II cytoskeletal 75	0.0129	3	35.9	94	123
K2C7	P08729	keratin type II cytoskeletal 7	0.0129	0	35.9	95	138
C1R	P00736	complement component 1 r subcomponent	0.0127	150	35.4	96	75
APOE	P02649	apolipoprotein E	0.0127	300	35.3	97	85
GGH	Q92820	gamma-glutamyl hydrolase	0.0126	10	35.1	98	125
CL43/COLEC10	P42916	collectin-43	0.0125	0	34.7	99	90
SERPINA3_7	A2I7N3	serpin A3-7	0.0120	0	33.4	100	104
PROS1	P07225	vitamin K-dependent protein	0.0119	100	33.0	101	63

		S					
F5	P12259	coagulation factor V	0.0113	15	31.5	102	101
C9	P02748	complement component C9	0.0109	200	30.5	103	82
APOD	P05090	apolipoprotein D	0.0106	200	29.5	104	80
KRT6B	P04259	keratin type II cytoskeletal 6B	0.0105	8	29.3	105	130
C4A	P0C0L4	complement C4-A	0.0103	200	28.8	106	91
GSN	P06396	gelsolin isoform a	0.0102	200	28.6	107	96
C5	P01031	complement C5a anaphylatoxin	0.0101	100	28.3	108	110
IGFALS	P35858	insulin-like growth factor-binding protein complex acid labile subunit	0.0098	80	27.4	109	119
RAB6B	Q14964	ras-related protein Rab-6B	0.0096	0	26.8	110	69
HBEGF	Q99075	proheparin-binding EGF-like growth factor	0.0096	0	26.8	111	92
FGG	P02679	fibrinogen gamma-B chain	0.0087	600	24.3	112	135
APOH	P02749	beta-2-glycoprotein 1	0.0087	800	24.2	113	112
CCDC76	Q9NUP7	tRNA guanosine-2'-O-methyltransferase TRM13 homolog	0.0083	0	23.2	114	121

ASPN	Q9BXN1	asporin	0.0081	0	22.6	115	105
ACTN1	P12814	alpha-actinin-1	0.0078	1	21.9	116	114
OIT3	Q8WWZ8	oncoprotein-induced transcript 3 protein	0.0073	3	20.4	117	117
COLEC10	Q9Y6Z7	collectin-10	0.0072	8	20.1	118	103
ACTN4	O43707	alpha-actinin-4	0.0066	6	18.4	119	133
FLNA	P21333	Filamin-A	0.0064	3	17.9	120	118
NUCB1	Q02818	nucleobindin-1	0.0063	10	17.6	121	140
AKR1C3	P42330	dihydrodiol dehydrogenase 3	0.0062	8	17.3	122	128
F9	P00740	coagulation factor IX	0.0062	40	17.2	123	77
COL6A3	P12111	collagen alpha-1(VI) chain	0.0058	1	16.3	124	132
YIPF3	Q9GZM5	protein YIPF3	0.0058	1	16.1	125	107
PKM2	P14618	pyruvate kinase isozymes M1/M2	0.0056	10	15.7	126	109
LAMB1	P07942	laminin subunit beta-1	0.0056	4	15.6	127	145
BGN	P21810	biglycan	0.0054	0	15.1	128	115
NSFL1C	Q9UNZ2	NSFL1 cofactor p47	0.0054	1	15.1	129	100
PRDX2	P32119	peroxiredoxin-2	0.0050	15	14.0	130	24
COL1A1	P02452	collagen alpha-1(XII) chain	0.0046	1	12.7	131	148
PDGFRB	P09619	beta-type platelet-derived growth factor receptor	0.0045	1	12.6	132	141
EFHC2	Q5JST6	EF-hand domain-containin	0.0044	0.5	12.4	133	126

		g family member C2					
ZBTB48	P10074	zinc finger and BTB domain-containing protein 48	0.0044	0	12.2	134	144
ELTD1	Q9HBW9	EGF latrophilin and seven transmembrane domain-containing protein 1	0.0044	1	12.1	135	161
ADAMTS13	Q76LX8	A disintegrin and metalloproteinase with thrombospondin motifs 13	0.0042	5	11.7	136	137
CEP290	O15078	centrosomal protein of 290 kDa	0.0041	0.01	11.5	137	162
GNL2	Q13823	nucleolar GTP-binding protein	0.0041	0	11.4	138	129
DARS	P14868	aspartyl-tRNA synthetase cytoplasmic	0.0040	0	11.1	139	120
SLC2A4	P14672	solute carrier family 2 facilitated glucose transporter member 4	0.0039	0	11.0	140	116
LRRN4	A4D1F6	leucine-rich repeat	0.0039	0	10.7	141	127

		transmembrane neuronal protein 4					
CDH6	P55285	cadherin-6	0.0038	0.8	10.6	142	150
TLR6	Q9Y2C9	toll-like receptor 6	0.0038	0	10.5	143	146
PLG	P00747	plasminogen	0.0037	400	10.3	144	139
SRC	P12931	v-src sarcoma (Schmidt-Ruppin A-2) viral oncogene homolog	0.0037	0	10.3	145	122
HSPD1	P10809	60 kDa heat shock protein mitochondrial	0.0035	1	9.7	146	111
CCDC147	Q5T655	coiled-coil domain-containing protein 147	0.0034	0	9.5	147	131
TMTC3	Q6ZXV5	transmembrane and TPR repeat-containing protein 3	0.0033	0	9.1	148	136
CYLC1	P35663	cylicin-1	0.0030	0	8.4	149	153
APP	P05067	amyloid beta A4 protein	0.0029	4	8.0	150	143
PIGG	Q5H8A4	GPI ethanolamine phosphate transferase 2	0.0029	0	8.0	151	157
LTF	P02788	lactotransferrin	0.0028	2	7.9	152	155
MMRN1	Q13201	multimerin-1	0.0028	7	7.7	153	163
HSPG2	P98160	basement	0.0027	2	7.6	154	156

		membrane-specific heparan sulfate proteoglycan core protein					
VCAM1	P19320	vascular cell adhesion molecule 1	0.0027	9	7.5	155	154
VCL	P18206	vinculin	0.0026	3	7.4	156	158
XP_874095_4	LOC616876	uncharacterized protein LOC616876 (~C1QC)	0.0025	100	7.1	157	134
AFG3L2	Q9Y4W6	AFG3-like protein 2	0.0025	0	6.9	158	152
NCAM1	P13591	neural cell adhesion molecule 1	0.0023	4	6.5	159	142
DNM2	P50570	dynamin-2	0.0023	0	6.4	160	147
ETAA1	Q9NY74	ewing's tumor-associated antigen 1 homolog	0.0022	0.3	6.2	161	149
NEBL	O76041	nebulette	0.0020	0	5.5	162	160
CSPG4	Q6UVK1	chondroitin sulfate proteoglycan 4	0.0017	0.2	4.8	163	166
ACAN	P16112	aggrecan core	0.0017	3	4.8	164	174
SMC1A	Q14683	structural maintenance of chromosomes protein1	0.0016	0	4.6	165	167
NRD1	O43847	nardilysin	0.0016	0	4.5	166	151
TLN2	Q9Y4G6	talin-2	0.0016	0.9	4.4	167	176
WDR17	Q8IZU2	WD repeat-containing protein	0.0016	0	4.3	168	159

		17					
TTLL5	Q6EMB2	tubulin polyglutamylase TTLL5 isoform 1	0.0016	0	4.3	169	168
CIT	O14578	citron Rho-interacting kinase	0.0014	0	4.0	170	165
NOTCH3	Q9UM47	neurogenic locus notch homolog protein 3-like	0.0013	0.5	3.6	171	173
NRXN2	P58401	neurexin-2-beta	0.0012	0	3.3	172	171
ARID2	Q68CP9	AT-rich interactive domain-containing protein 2	0.0011	0	3.0	173	175
CEP350	Q5VT06	centrosome-associated protein 350 isoform 1	0.0010	0.1	2.7	174	169
MYH9	P35579	myosin-IXb	0.0009	1	2.6	175	170
FLNC	Q14315	filamin-C	0.0007	0.7	2.1	176	172
DNAH8	Q96JB1	dynein heavy chain 2 axonemal	0.0005	0.07	1.3	177	177
TTN	Q8WZ42	titin	0.0004	0.07	1.0	178	178

Supplementary Figure 3. Linear regression analysis between the logarithm of plasma concentration (nmol) and the logarithm of relative abundance as obtained by mass spectrometry data. Regression analysis at a 95% confidence level showed a linear correlation between protein abundance in mass spectrometry and the concentration in plasma ($r=0.66$, $p < 0.0001$).

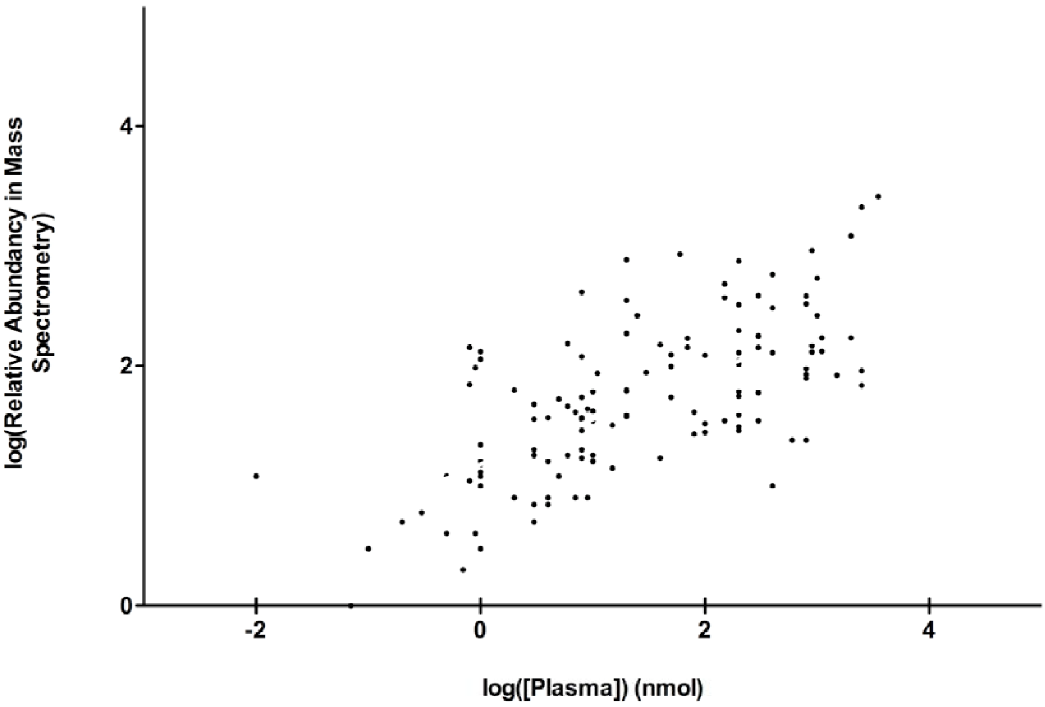

Supplementary Table 2. **Relative abundance at the domain level**. A list of selected frequently appearing InterPro domains (IP number & IP name), their function as accepted and annotated by Gene Ontology, the number of different proteins sharing them, and their mean of relative abundance.

IP_Number	IP_Name	Protein function (Gene Ontology)	Number	Mean of domain RA
"IPR000215"	Protease_inhib_I4_serpin	serine-type endopeptidase inhibitor activity (GO)	13	51
"IPR002035"	VWF_A	protein binding (GO)	6	53
"IPR001599"	Macroglobln_a2	endopeptidase inhibitor activity (GO)	5	59
"IPR018039"	Intermediate_filament_CS	cytoskeleton structure	7	71
"IPR016060"	Complement_control_module	protein binding	9	75
"IPR013806"	Kringle-like	regulation of proteolytic activity	5	77
"IPR009003"	Pept_cys/ser_Trypsin-like	(protease) catalytic activity (GO)	7	78
"IPR003591"	Leu-rich_rpt_typical-subtyp	LRR proteins	6	78
"IPR000372"	LRR-contain_N	N-terminal LRR	7	82
"IPR001254"	Peptidase_S1_S6	serine-type endopeptidase activity (GO)	9	84
"IPR000859"	CUB	MEROPS peptidase	5	91
"IPR001881"	EGF-like_Ca-bd	calcium ion binding (GO)	14	92
"IPR001611"	Leu-rich_rpt	LRR proteins	9	95
"IPR011992"	EF-hand-like_dom	calcium ion binding (GO)	6	97
"IPR013032"	EGF-like_reg_C	-	17	98
"IPR006210"	EGF-like	protein binding (GO)	18	107
"IPR008985"	ConA-like_lec_gl	adhesion	8	108
"IPR013320"	ConA-like_subgrp	lectin	8	119
"IPR008160"	Collagen	adhesion	5	125
"IPR006209"	EGF	protein binding (GO)	9	126
"IPR013783"	Ig-like_fold	-	12	127
"IPR011993"	PH_type	signalling	5	127
"IPR007110"	Ig-like	protein binding (GO)	8	130
"IPR003961"	Fibronectin_type3	protein binding(GO)	5	141

"IPR013098"	Ig_I-set	adhesion	5	155

Supplementary Figure 4. Interactions of purified gene products at the action level as retrieved from String. A total of 178 identified genes were submitted to the STRING database (http://string-db.org/). Of these, 169 genes were recognized by the STRING database and more than 80% showed interactions, either at the evidence, confidence, or action level.

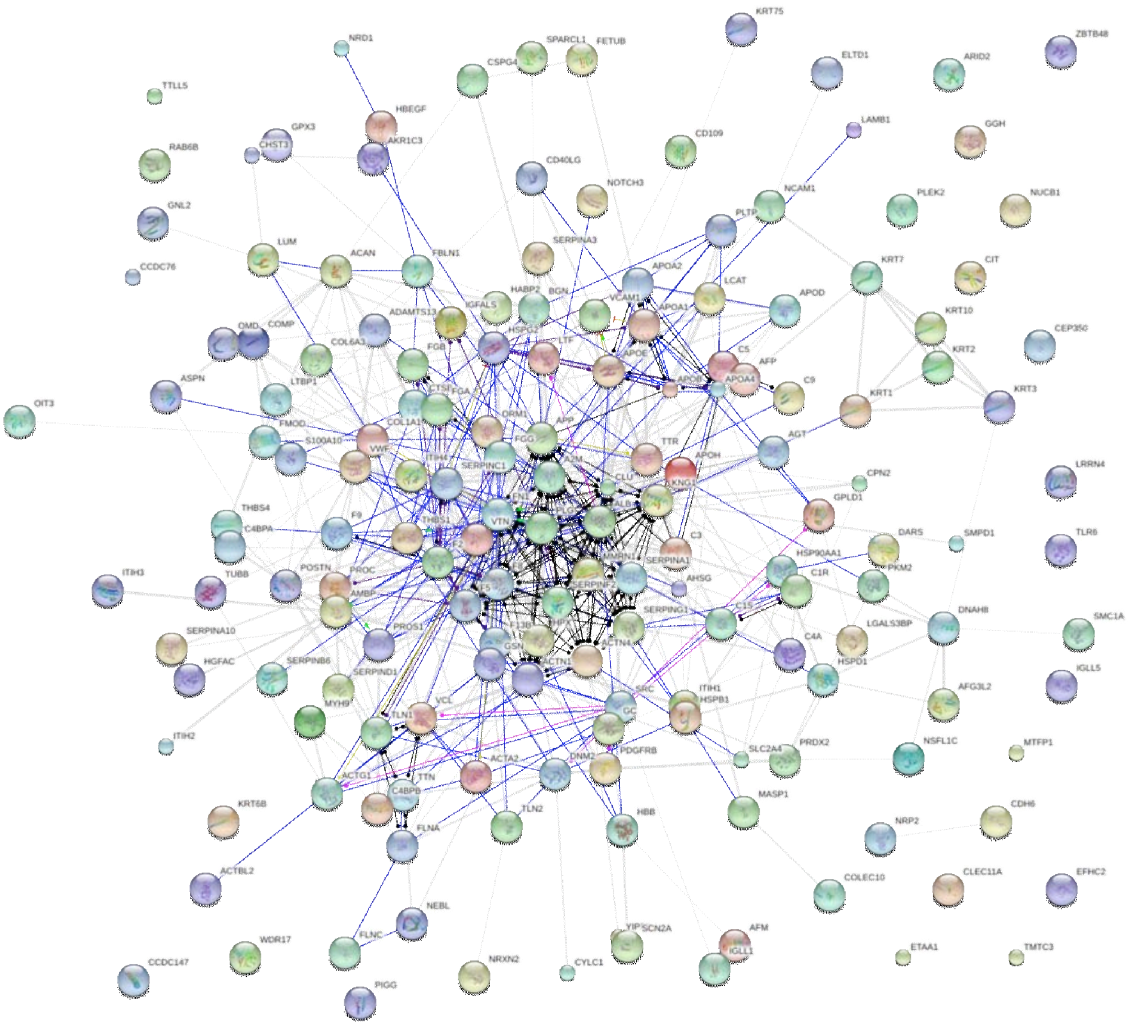

Supplementary Table 3[2]. List of significantly upregulated genes in HMEC 184 cells (90% confluence) following 24h exposure to ENPs (25 µg/mL) identified with DAVID Genecard DB. Gene, description, breast expression, fold change as compared to control (FC), and p-value.

**Gene or biological process**	**Description of gene or Gene Ontology**	**Breast Expres-sion***	**FC**	***p value***
	Inflammatory response (GO:0006954)			
PLA2G4D	phospholipase A2_group IVD	1	5.44	7.22E-05
ELF3	E74-like factor 3 (ets domain transcription factor_epithelial-specific)	43	4.07	1.06E-04
PLBD1	phospholipase B domain containing 1	33	3.07	1.42E-04
IL36RN	interleukin 36 receptor antagonist	0	2.96	1.06E-04
HPGD	hydroxyprostaglandin dehydrogenase 15-(NAD)	11	2.90	2.72E-06
IL1RN	interleukin 1 receptor antagonist	38	2.66	4.61E-05
SAA3P	serum amyloid A3 pseudogene	11	2.61	4.78E-03
IL36G	interleukin 36_gamma	0	2.60	3.32E-04
HMOX1	heme oxygenase (decycling) 1	38	2.57	5.51E-04
S100A12	S100 calcium binding protein A12	4	2.44	6.45E-05
IL8	interleukin 8	16	2.20	3.64E-04
TLR1	toll-like receptor 1	14	2.15	1.43E-03
PLA2G7	phospholipase A2_group VII (platelet-activating factor acetylhydrolase_plasma)	19	2.15	6.46E-04
ANXA1	annexin A1	285	2.12	5.10E-05
TLR2	toll-like receptor 2	13	2.05	5.03E-04
	Cell junction (GO:0005911)			
CLDN4	claudin 4	49	6.07	6.21E-06
CLDN3	claudin 3	23	3.61	8.05E-05
CGN	cingulin	14	2.95	2.85E-04
CLDN7	claudin 7	20	2.62	1.49E-04
DSG4	desmoglein 4	2	2.22	2.09E-05
CDSN	corneodesmosin	0	2.18	6.43E-04
	Cell-Cell adhesion (GO:0016337)			
PCDH1	protocadherin 1	28	2.83	1.28E-05
CEACAM6	carcinoembryonic antigen-related cell adhesion molecule 6	4	2.78	1.16E-04
CEACAM1	carcinoembryonic antigen-related cell adhesion molecule 1 (biliary glycoprotein)	20	2.13	3.25E-04
ADAM8	ADAM metallopeptidase domain 8	7	2.09	6.57E-04
Cellular process : Biosynthesis				
	Glycosphingolipid biosynthesis (hsa00601#)			

[2] *: in Illumina Body Map (100xFPKM$^{1/2}$, Fragments Per Kilobase of exon per Million fragments mapped were calculated using the Cufflinks program and thereupon rescaled by multiplying FPKM by 100 and then calculating the root); **#**: has a gene ID retrieved from www.genome.jp/kegg/

FUT2	fucosyltransferase 2 (secretor status included)	4	3.02	4.40E-04
B3GNT3	UDP-GlcNAc:betaGal beta-1.3-N-acetylglucosaminyltransferase 3	13	2.71	3.42E-05
FUT3	fucosyltransferase 3 (galactoside 3(4)-L-fucosyltransferase_Lewis blood group)	3	2.57	4.37E-05
	Glycoprotein biosynthetic process (GO:0009101)			
B3GALT4	UDP-Gal:betaGlcNAc beta 1.3-galactosyltransferase_polypeptide 4	20	3.06	8.81E-05
	Glycerolipid biosynthetic process (GO:0045017)			
CWH43	hypothetical protein FLJ21511	0	3.08	1.06E-04
DGAT2	diacylglycerol O-acyltransferase homolog 2 (mouse)	215	2.75	8.90E-04
AGPAT9	1-acylglycerol-3-phosphate O-acyltransferase 9	16	2.47	2.50E-04
	Lipid biosynthetic process (GO:0008610)			
FA2H	fatty acid 2-hydroxylase	6	2.97	8.49E-05
ELOVL4	elongation of very long chain fatty acids (FEN1/Elo2_SUR4/Elo3_yeast)-like 4	7	2.09	3.75E-04
	purine nucleotide biosynthetic process (GO:0006164)			
ATP12A	ATPase_ H^+/K^+ transporting_nongastric_alpha polypeptide	0	5.85	5.71E-06
GUCY1A3	guanylate cyclase 1_soluble_alpha 3	29	2.66	1.62E-05
	ATP binding (GO:0005524)			
PRR15L	ATPase family_AAA domain containing 4	12	5.14	2.94E-05
OASL	2'-5'-oligoadenylate synthetase-like	10	2.88	2.36E-04
ERBB3	v-erb-b2 erythroblastic leukemia viral oncogene homolog 3 (avian)	31	2.71	9.35E-05
NLRP10	NLR family_pyrin domain containing 10	0	2.49	5.86E-05
PIM1	pim-1 oncogene	44	2.06	1.51E-04
KIF13B	kinesin family member 13B	20	2.00	3.44E-06
	Ribosome biogenesis			
ISG20	interferon stimulated exonuclease gene 20kDa	22	2.70	3.49E-07
	Angiogenesis (GO:0001525)			
S100A7	S100 calcium binding protein A7	4	3.12	9.75E-04
CEACAM1	carcinoembryonic antigen-related cell adhesion molecule 1 (biliary glycoprotein)	20	2.13	3.25E-04
Cellular process : Proliferation				
	Regulation of cell proliferation (GO:0042127)			
CAPN14	calpain 14	2	22.87	9.73E-09
S100P	S100 calcium binding protein P	11	7.74	4.01E-06
VTCN1	V-set domain containing T cell activation inhibitor 1	25	5.29	3.93E-05
LIPH	lipase_member H	8	4.99	2.27E-06
CDKN1C	cyclin-dependent kinase inhibitor 1C (p57_Kip2)	63	4.48	5.36E-05
RASSF3	Ras association (RalGDS/AF-6) domain family member 3	61	3.99	2.88E-04
RTKN2	rhotekin 2	4	2.65	5.06E-05
MARCKSL1	MARCKS-like 1	49	2.48	2.70E-04
RARRES1	retinoic acid receptor responder (tazarotene induced) 1	24	2.38	1.19E-04
CAPN5	calpain 5	13	2.21	2.24E-05
IGFL2	IGF-like family member 2	0	2.09	2.34E-05

KLK6	kallikrein-related peptidase 6	11	2.06	1.83E-04
	Proliferation : Wnt signaling pathway			
NDRG2	NDRG family member 2	118	2.16	5.48E-04
APCDD1	adenomatosis polyposis coli down-regulated 1	46	2.09	6.51E-04
	Cell cycle phase (GO:0022403)			
MAP2	microtubule-associated protein 2	16	2.77	8.53E-05
MAPRE2	microtubule-associated protein_RP/EB family_member 2	33	2.45	1.34E-04
OVOL1	ovo-like 1(Drosophila)	4	2.31	9.03E-05
Transcription				
	Regulation of transcription_DNA-dependent (GO:0006355)			
HOPX	HOP homeobox	13	3.25	4.73E-04
	Regulation of transcription (GO:0006355)			
MACC1	metastasis associated in colon cancer 1	8	3.00	5.63E-04
Cell structure				
	cytoskeletal part (GO:0044430)			
KRT23	keratin 23 (histone deacetylase inducible)	29	8.59	2.04E-04
KRT80	keratin 80	11	6.64	6.43E-07
KRT34	keratin 34	0	2.96	4.23E-04
KRT24	keratin 24	1	2.55	1.51E-04
FLG	filaggrin	2	2.27	3.49E-04
	Golgi membrane (GO:0000139)			
CHST6	carbohydrate (N-acetylglucosamine 6-O) sulfotransferase 6	1	2.96	9.72E-04
MALL	mal_T-cell differentiation protein-like	33	2.68	7.61E-05
	Organelle membrane (GO:0031090)			
RAB11FIP1	RAB11 family interacting protein 1 (class I)	23	4.60	7.30E-05
CYP4B1	cytochrome P450_family 4_subfamily B_polypeptide 1	26	3.34	4.60E-05
SPINK5	serine peptidase inhibitor_Kazal type 5	5	2.58	7.79E-04
Cellular process : cell death and apoptosis				
	Negative regulation of apoptosis (GO:0043066)			
SERPINB2	serpin peptidase inhibitor_clade B (ovalbumin)_member 2	9	3.48	3.63E-04
	Regulation of apoptosis (GO:0042981)			
MMP10	matrix metallopeptidase 10	2	3.54	7.88E-05
MMP1	matrix metallopeptidase 1	0	3.18	4.66E-04
MMP9	matrix metallopeptidase 9 (gelatinase B_92kDa gelatinase_92kDa type IV collagenase)	39	2.06	7.62E-04
	Cell death			
BNIPL	BCL2/adenovirus E1B 19kD interacting protein like	12	3.19	5.63E-06
LCN2	lipocalin 2	18	2.30	6.47E-06
Endocytosis and trafficking				

RAB11FIP1	RAB11 family interacting protein 1 (class I)	23	4.60	7.30E-05
MARCKSL 1	MARCKS-like 1	49	2.48	2.70E-04
MARCO	macrophage receptor with collagenous structure	46	2.11	3.03E-04
SPON2	spondin 2_extracellular matrix protein	46	2.06	7.42E-04
MYO5B	myosin VB	13	2.05	3.03E-05
MAL2	mal_T-cell differentiation protein 2 (gene/pseudogene)	68	2.01	2.90E-04
Oxydant stress				
NCF2	neutrophil cytosolic factor 2	30	3.61	8.57E-05
GGT6	gamma-glutamyltransferase 6	11	2.00	1.34E-04
Miscellaneous				
MUC16	mucin 16_cell surface associated	9	2.70	1.09E-04

Supplementary Table 4[3]. Functional annotation analysis of microarry data sets using ADVID. GO terms were significantly enriched in genes at least twofold upregulated in HMEC 184 cells (90% confluence) in response to 24h exposure to 25 µg/mL ENPs.

Cluster *	Category	Term	Count	P value#	FE
Cluster 1	GOTERM_CC_FAT	cornified envelope	9	1.78E-11	41.83
	SP_PIR_KEYWORDS	keratinization	7	6.27E-07	22.29
	GOTERM_BP_FAT	keratinocyte differentiation	12	8.22E-12	21.02
	GOTERM_BP_FAT	epidermal cell differentiation	13	8.83E-13	20.88
	GOTERM_BP_FAT	keratinization	7	1.64E-06	18.82
	GOTERM_BP_FAT	peptide cross-linking	4	1.38E-03	17.79
	GOTERM_BP_FAT	epithelial cell differentiation	16	6.41E-13	13.50
	GOTERM_BP_FAT	epidermis development	18	2.81E-13	11.31
	GOTERM_BP_FAT	ectoderm development	18	1.02E-12	10.46
	GOTERM_BP_FAT	epithelium development	16	9.11E-10	8.15
Cluster 2	SP_PIR_KEYWORDS	inflammatory response	5	3.09E-03	8.27
	GOTERM_BP_FAT	inflammatory response	10	1.91E-03	3.56
	GOTERM_BP_FAT	response to wounding	15	1.74E-04	3.27
	GOTERM_BP_FAT	defense response	12	1.64E-02	2.26
Cluster 3	INTERPRO	keratin_type I	3	2.80E-02	11.39
	INTERPRO	filament	4	1.95E-02	6.96
	INTERPRO	intermediate filament protein_conserved site	4	1.95E-02	6.96
	GOTERM_MF_FAT	structural molecule activity	14	1.24E-03	2.81

[3] *: Biological process (BP), cellular component (CC), and molecular function (MF) in Gene Ontology (GO), single protein of protein information ressource (SP_PIR), protein domains or sites (INTERPRO) and pathway extracted from Kyoto Encyclopedia of Genes and Genomes (KEGG); #: or EASE score, modified Fisher's exact test according to DAVID software cut-off

Cluster 4	GOTERM_CC_FAT	desmosome	3	1.58E-02	15.34
	SP_PIR_KEYWORDS	tight junction	3	8.21E-02	6.26
	GOTERM_CC_FAT	apical junction complex	6	2.74E-03	6.20
	GOTERM_CC_FAT	apicolateral plasma membrane	6	3.12E-03	6.02
	GOTERM_CC_FAT	cell-cell junction	8	2.50E-03	4.31
Cluster 5	SP_PIR_KEYWORDS	inflammatory response	5	3.09E-03	8.27
Cluster 6	KEGG_PATHWAY	glycosphingolipid biosynthesis	3	1.12E-02	17.95
	SP_PIR_KEYWORDS	signal-anchor	10	5.85E-03	3.03
Cluster 7	GOTERM_BP_FAT	glycerolipid biosynthetic process	4	3.13E-02	5.78
Cluster 8	GOTERM_BP_FAT	wound healing	6	2.43E-02	3.63
